# Supplementary material for: Data-driven discovery of changes in clinical code usage over time: a case-study on changes in cardiovascular disease recording in two English electronic health records databases (2001–2015)
Source: BMJ Open. 2020 Feb 13;10(2):e034396. doi: 10.1136/bmjopen-2019-034396 (PMC7045100; doi:10.1136/bmjopen-2019-034396)
Supplement: Supplementary data [file bmjopen-2019-034396supp001.pdf]

Supplementary material

Manuscript: Data-driven discovery of changes in clinical code usage over time: a case-study on changes in cardiovascular disease recording in two English electronic health records databases (2001-2015)

Supplementary Table 1 - Read codes taken from the Quality and Outcomes Framework (QOF) used to identify history of cardiovascular disease

CHD codes	Description
G3...00	Ischaemic heart disease
G30..00	Acute myocardial infarction
G300.00	Acute anterolateral infarction
G301.00	Other specified anterior myocardial infarction
G301000	Acute anteroapical infarction
G301100	Acute anteroseptal infarction
G301z00	Anterior myocardial infarction NOS
G302.00	Acute inferolateral infarction
G303.00	Acute inferoposterior infarction
G304.00	Posterior myocardial infarction NOS
G305.00	Lateral myocardial infarction NOS
G306.00	True posterior myocardial infarction
G307.00	Acute subendocardial infarction
G307000	Acute non-Q wave infarction
G307100	Acute non-ST segment elevation myocardial infarction
G308.00	Inferior myocardial infarction NOS
G309.00	Acute Q-wave infarct
G30B.00	Acute posterolateral myocardial infarction
G30X.00	Acute transmural myocardial infarction of unspecified site
G30X000	Acute ST segment elevation myocardial infarction
G30y.00	Other acute myocardial infarction
G30y000	Acute atrial infarction
G30y100	Acute papillary muscle infarction
G30y200	Acute septal infarction
G30yz00	Other acute myocardial infarction NOS
G30z.00	Acute myocardial infarction NOS
G31..00	Other acute and subacute ischaemic heart disease
G311.00	Preinfarction syndrome
G311000	Myocardial infarction aborted

G311100	Unstable angina
G311200	Angina at rest
G311300	Refractory angina
G311400	Worsening angina
G311500	Acute coronary syndrome
G311z00	Preinfarction syndrome NOS
G312.00	Coronary thrombosis not resulting in myocardial infarction
G31y.00	Other acute and subacute ischaemic heart disease
G31y000	Acute coronary insufficiency
G31y100	Microinfarction of heart
G31y200	Subendocardial ischaemia
G31y300	Transient myocardial ischaemia
G31yz00	Other acute and subacute ischaemic heart disease NOS
G32..00	Old myocardial infarction
G33..00	Angina pectoris
G330.00	Angina decubitus
G330000	Nocturnal angina
G330z00	Angina decubitus NOS
G33z.00	Angina pectoris NOS
G33z000	Status anginosus
G33z100	Stenocardia
G33z200	Syncope anginosa
G33z300	Angina on effort
G33z400	Ischaemic chest pain
G33z500	Post infarct angina
G33z600	New onset angina
G33z700	Stable angina
G33zz00	Angina pectoris NOS
G34..00	Other chronic ischaemic heart disease
G340.00	Coronary atherosclerosis
G340000	Single coronary vessel disease
G340100	Double coronary vessel disease
G342.00	Atherosclerotic cardiovascular disease
G343.00	Ischaemic cardiomyopathy
G344.00	Silent myocardial ischaemia
G34y.00	Other specified chronic ischaemic heart disease
G34y000	Chronic coronary insufficiency
G34y100	Chronic myocardial ischaemia
G34yz00	Other specified chronic ischaemic heart disease NOS
G34z.00	Other chronic ischaemic heart disease NOS

G34z000	Asymptomatic coronary heart disease
G35..00	Subsequent myocardial infarction
G350.00	Subsequent myocardial infarction of anterior wall
G351.00	Subsequent myocardial infarction of inferior wall
G353.00	Subsequent myocardial infarction of other sites
G35X.00	Subsequent myocardial infarction of unspecified site
G38..00	Postoperative myocardial infarction
G380.00	Postoperative transmural myocardial infarction of anterior wall
G381.00	Postoperative transmural myocardial infarction of inferior wall
G382.00	Postoperative transmural myocardial infarction of other sites
G383.00	Postoperative transmural myocardial infarction of unspecified site
G384.00	Postoperative subendocardial myocardial infarction
G38z.00	Postoperative myocardial infarction, unspecified
G39..00	Coronary microvascular disease
G3y..00	Other specified ischaemic heart disease
G3z..00	Ischaemic heart disease NOS
Gyu3.00	[X]Ischaemic heart diseases
Gyu3000	[X]Other forms of angina pectoris
Gyu3200	[X]Other forms of acute ischaemic heart disease
Gyu3300	[X]Other forms of chronic ischaemic heart disease
Gyu3400	[X]Acute transmural myocardial infarction of unspecified site
Gyu3500	[X]Subsequent myocardial infarction of other sites
Gyu3600	[X]Subsequent myocardial infarction of unspecified site
**Heart failure codes**	**Description**
662f.	New York Heart Association classification - class I
662g.	New York Heart Association classification - class II
662h.	New York Heart Association classification - class III
662i.	New York Heart Association classification - class IV
G1yz1	Rheumatic left ventricular failure
G58..	Heart failure
G580.	Congestive heart failure
G5800	Acute congestive heart failure
G5801	Chronic congestive heart failure
G5802	Decompensated cardiac failure
G5803	Compensated cardiac failure
G5804	Congestive heart failure due to valvular disease
G581.	Left ventricular failure
G5810	Acute left ventricular failure
G582.	Acute heart failure

G583.	Heart failure with normal ejection fraction
G584.	Right ventricular failure
G58z.	Heart failure NOS
**PAD codes**	**Description**
G73..	Other peripheral vascular disease
G734.	Peripheral arterial disease
G73y.	Other specified peripheral vascular disease
G73z.	Peripheral vascular disease NOS
G73z0	Intermittent claudication
G73zz	Peripheral vascular disease NOS
Gyu74	[X]Other specified peripheral vascular diseases
**Stroke codes**	**Description**
Fyu55	[X]Other transient cerebral ischaemic attacks and related syndromes
G61..	Intracerebral haemorrhage
G610.	Cortical haemorrhage
G611.	Internal capsule haemorrhage
G612.	Basal nucleus haemorrhage
G613.	Cerebellar haemorrhage
G614.	Pontine haemorrhage
G615.	Bulbar haemorrhage
G616.	External capsule haemorrhage
G618.	Intracerebral haemorrhage, multiple localized
G619.	Lobar cerebral haemorrhage
G61X.	Intracerebral haemorrhage in hemisphere, unspecified
G61X0	Left sided intracerebral haemorrhage, unspecified
G61X1	Right sided intracerebral haemorrhage, unspecified
G61z.	Intracerebral haemorrhage NOS
G63y0	Cerebral infarct due to thrombosis of precerebral arteries
G63y1	Cerebral infarction due to embolism of precerebral arteries
G64..	Cerebral arterial occlusion
G640.	Cerebral thrombosis
G6400	Cerebral infarction due to thrombosis of cerebral arteries
G641.	Cerebral embolism
G6410	Cerebral infarction due to embolism of cerebral arteries
G64z.	Cerebral infarction NOS
G64z0	Brainstem infarction
G64z1	Wallenberg syndrome
G64z2	Left sided cerebral infarction

G64z3	Right sided cerebral infarction
G64z4	Infarction of basal ganglia
G65..	Transient cerebral ischaemia
G650.	Basilar artery syndrome
G651.	Vertebral artery syndrome
G6510	Vertebro-basilar artery syndrome
G652.	Subclavian steal syndrome
G653.	Carotid artery syndrome hemispheric
G654.	Multiple and bilateral precerebral artery syndromes
G656.	Vertebrobasilar insufficiency
G657.	Carotid territory transient ischaemic attack
G65y.	Other transient cerebral ischaemia
G65z.	Transient cerebral ischaemia NOS
G65z0	Impending cerebral ischaemia
G65z1	Intermittent cerebral ischaemia
G65zz	Transient cerebral ischaemia NOS
G66..	Stroke and cerebrovascular accident unspecified
G660.	Middle cerebral artery syndrome
G661.	Anterior cerebral artery syndrome
G662.	Posterior cerebral artery syndrome
G663.	Brain stem stroke syndrome
G664.	Cerebellar stroke syndrome
G665.	Pure motor lacunar syndrome
G666.	Pure sensory lacunar syndrome
G667.	Left sided CVA
G668.	Right sided CVA
G6760	Cerebral infarction due to cerebral venous thrombosis, nonpyogenic
G6W..	Cerebral infarction due to unspecified occlusion or stenosis of precerebral arteries
G6X..	Cerebral infarction due to unspecified occlusion or stenosis of cerebral arteries
Gyu62	[X]Other intracerebral haemorrhage
Gyu63	[X]Cerebral infarction due to unspecified occlusion or stenosis of cerebral arteries
Gyu64	[X]Other cerebral infarction
Gyu65	[X]Occlusion and stenosis of other precerebral arteries
Gyu66	[X]Occlusion and stenosis of other cerebral arteries
Gyu6F	[X]Intracerebral haemorrhage in hemisphere, unspecified
Gyu6G	[X]Cerebral infarction due to unspecified occlusion or stenosis of precerebral arteries
ZV12D	[V]Personal history of transient ischaemic attack

Supplementary Table 2 - ICD-10 chapters used to identify admissions for cardiovascular disease

ICD-10 chapter	Description
I20	Angina pectoris
I21	Acute myocardial infarction
I22	Subsequent myocardial infarction
I23	Certain current complications following acute myocardial infarction
I24	Other acute ischaemic heart diseases
I25	Chronic ischaemic heart disease
I26	Pulmonary embolism
I27	Other pulmonary heart diseases
I28	Other diseases of pulmonary vessels
I30	Acute pericarditis
I31	Other diseases of pericardium
I32	Pericarditis in diseases classified elsewhere
I33	Acute and subacute endocarditis
I34	Nonrheumatic mitral valve disorders
I35	Nonrheumatic aortic valve disorders
I36	Nonrheumatic tricuspid valve disorders
I37	Pulmonary valve disorders
I38	Endocarditis, valve unspecified
I39	Endocarditis and heart valve disorders in diseases classified elsewhere
I40	Acute myocarditis
I41	Myocarditis in diseases classified elsewhere
I42	Cardiomyopathy
I43	Cardiomyopathy in diseases classified elsewhere
I44	Atrioventricular and left bundle-branch block
I45	Other conduction disorders
I46	Cardiac arrest
I47	Paroxysmal tachycardia
I48	Atrial fibrillation and flutter
I49	Other cardiac arrhythmias
I50	Heart failure
I51	Complications and ill-defined descriptions of heart disease
I52	Other heart disorders in diseases classified elsewhere
I60	Subarachnoid haemorrhage
I61	Intracerebral haemorrhage
I62	Other nontraumatic intracranial haemorrhage
I63	Cerebral infarction
I64	Stroke, not specified as haemorrhage or infarction

I65	Occlusion and stenosis of precerebral arteries, not resulting in cerebral infarction
I66	Occlusion and stenosis of cerebral arteries, not resulting in cerebral infarction
I67	Other cerebrovascular diseases
I68	Cerebrovascular disorders in diseases classified elsewhere
I69	Sequelae of cerebrovascular disease

Supplementary Table 3 - Variables included in the CPRD analysis

**Variable**	**Categories**
Age	20-39, 40-59, 60-79, ≥80
Sex	Male, Female
Index of Multiple Deprivation	Quintiles
Coronary heart disease	Yes (at anytime in medical history), No
Heart failure	Yes (at anytime in medical history), No
Peripheral arterial disease	(at anytime in medical history)
Stroke	Stroke (at anytime in medical history), Transient Ischaemic Attack (at anytime in medical history but no stroke), No

Supplementary Table 4 - Univariable changes over time in CPRD (by mid-year)

	**2001**	**2005**	**2010**	**2015**
**Number of patients**	1,122,722	1,840,798	2,207,027	1,340,441
**Age (%)**				
20-39	31.6	31.2	31.9	31.5
40-59	41.1	38.9	36.8	36.8
60-79	23.8	24.2	24.5	24.8
≥80	3.4	5.7	6.7	6.9
**Female (%)**	50.9	50.6	50.6	50.8
**Index of Multiple Deprivation (mean)**	2.72	2.74	2.75	2.75
**Coronary heart disease (%)**	4.5	4.9	4.5	4.0
**Heart failure (%)**	0.7	1.0	1.0	1.0
**Peripheral arterial disease (%)**	0.7	1.0	1.0	0.8

Stroke (%)				
Transient ischaemic attack	0.7	0.9	1.0	0.9
Stroke	0.8	1.2	1.4	1.4

Note: Data on Index of Multiple Deprivation was missing for <0.1% of patients. These patients were excluded from the analysis.

Supplementary Table 5 - Variables included in the HES analysis

Variable	Categories
Age	40-44, 45-49, 50-54, 55-59, 60-64, 65-69, 70-74, ≥75
Sex	Male, Female
Index of Multiple Deprivation	Quintiles
Cardiovascular code	3-character ICD-10 code ranging from I20 to I69

Note: admissions with more than one included cardiovascular code were counted multiple times

Supplementary Table 6 - Univariable changes over time in HES (by mid-year)

	2001	2005	2010	2015
**Number of admissions**	38861	38855	39318	42756
**Age (%)**				
40-44	3.06	3.76	3.31	2.85
45-49	4.79	5.46	5.66	5.26
50-54	8.74	7.75	8.09	8.47
55-59	11.29	11.66	10.53	11.01
60-64	13.93	13.73	15.33	13.02
65-69	16.76	16.66	16.07	18.19
70-74	18.85	18.70	18.71	18.23
≥75	22.58	22.25	22.27	22.92
**Female (%)**	36.21	35.95	35.66	35.26
**Index of Multiple Deprivation (mean)**	1.74	1.79	1.77	1.76
**Cardiovascular code (top 5 %)**				
I25 Chronic CHD	23.4	26.52	27.39	22.94

I20 Angina pectoris	19.8	15.74	11.97	7.75
I48 Atrial fibrillation	11.29	12.72	15.56	16.85
I21 Acute myocardial infarction	10.53	10.21	6.85	11.40
I50 Heart failure	8.33	6.81	5.98	7.39

Note: There was no missing data

Supplementary Figure 1 - Number of primary care practice included in the analysis by month

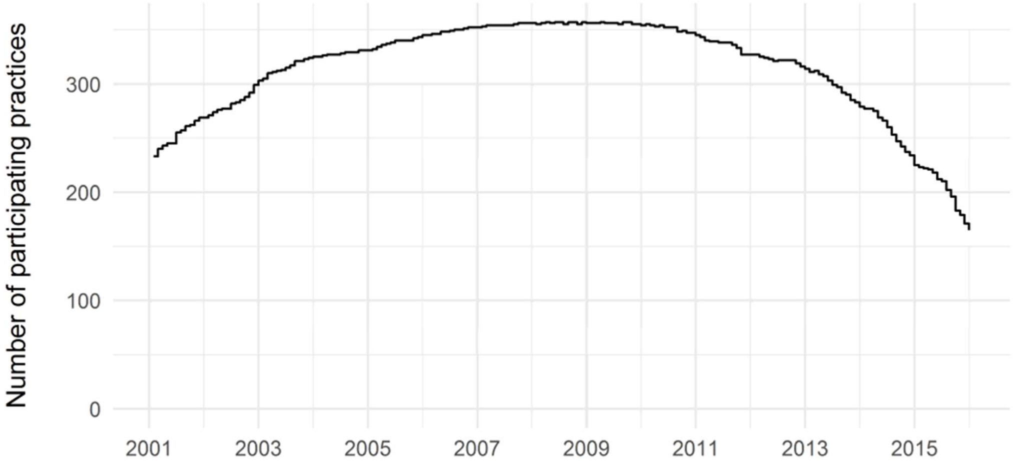

Supplementary Figure 2 - Information-Geometric Temporal (IGT) plot of demography (including age) and cardiovascular disease prevalence in CPRD between 2001 and 2015. The observed vertical columns are due to the way CPRD records age (birth year only), so changes in age can only happen in July.

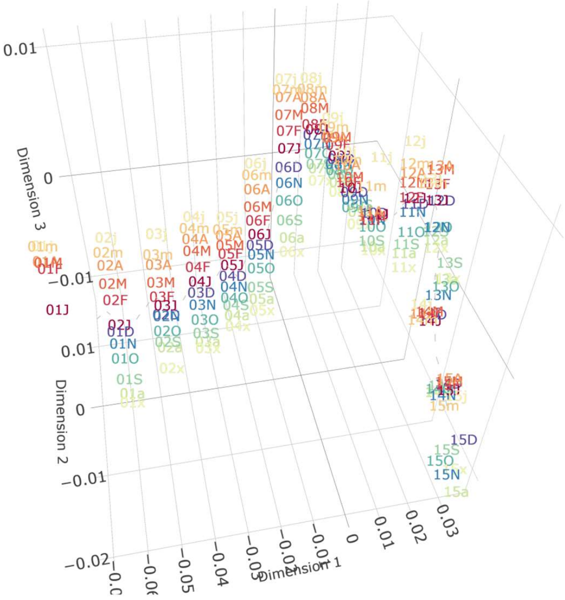

Supplementary Figure 3 - Information-Geometric Temporal (IGT) subplots of Figure 2
(a) Between 2001-2008 there was a gradual increase in disease prevalence, with two indentations corresponding to the years 2003 and 2005. (b) In 2008, the general trend reverses and prevalences decrease again, shown by a change in the direction of the graph. (c) The magnitude of variability increases after 2011, predominantly owing to changes in the socio-economic status due to a reduction in the number of practices contributing to the dataset.

(a)

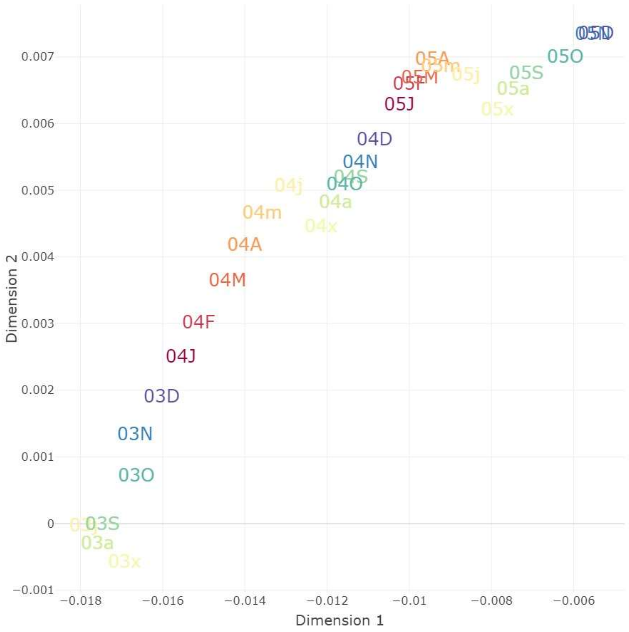

(b)

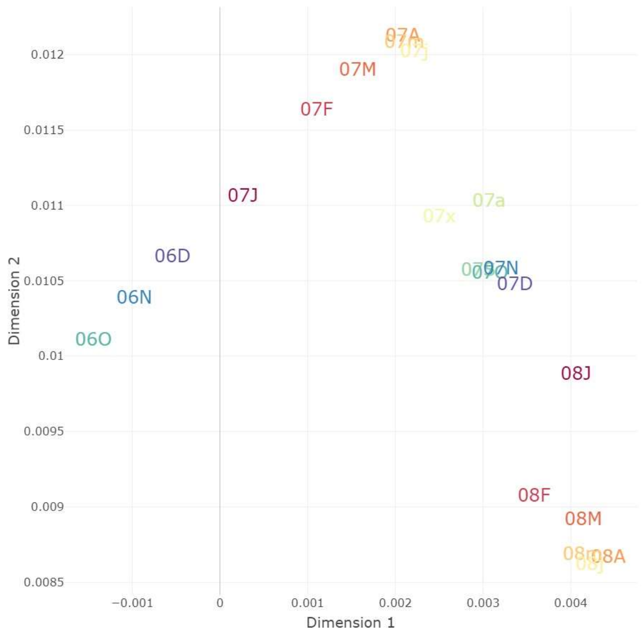
Dimension 1
Dimension 2
07A
07M
07F
07J
07a
07x
06D
07N
07D
06N
06O
08J
08F
08M
08A

(c)

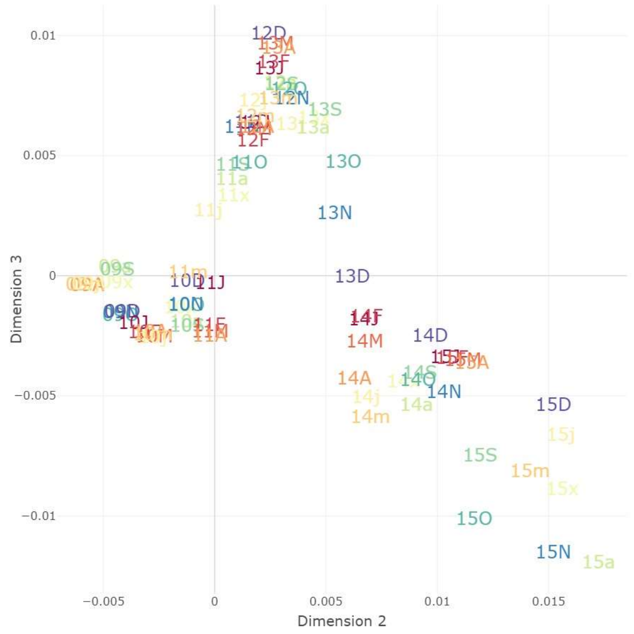
Dimension 2
Dimension 3
12D
13S
13O
13N
13D
11O
11a
11x
11j
12F
09S
10D
11J
10N
14D
14M
14A
14N
14a
14m
15D
15j
15S
15m
15x
15O
15N
15a

Supplementary Figure 4 - Information-Geometric Temporal (IGT) subplots of Figure 3
(a) From 2001-2009 there was a gradual change in which cardiovascular codes were associated with hospital admission. The data distributions starts to diverge from the previous trend in March 2009. (b) In March 2010, the distribution of cardiovascular codes abruptly changes. (c and d) Similar and even stronger changes in cardiovascular disease coding occurred again in April 2012 and April 2014.

(a)

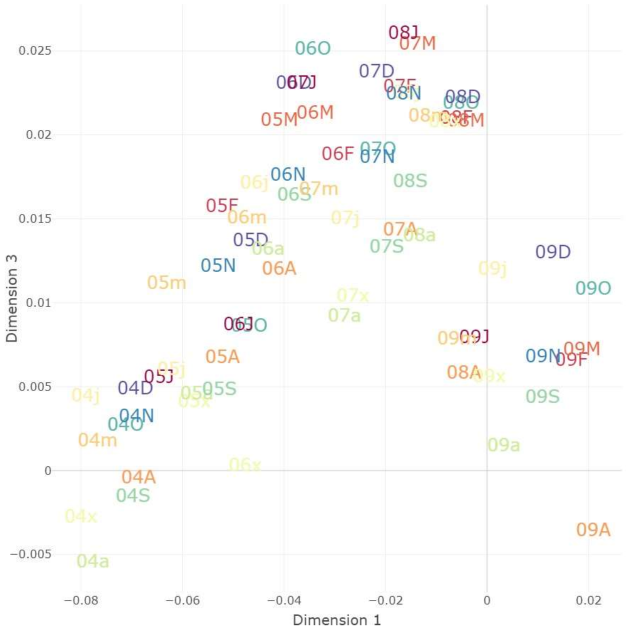

(b)

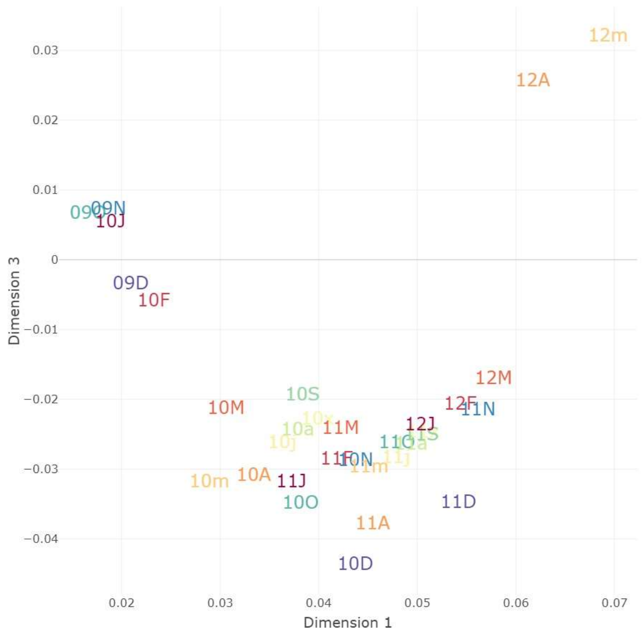
Dimension 3
Dimension 1
12m
12A
09N
10J
09D
10F
12M
10S
10M
12F
11N
11M
12J
10a
10j
11O
11F
10N
11j
11m
10A
10m
11J
10O
11D
11A
10D

(c)

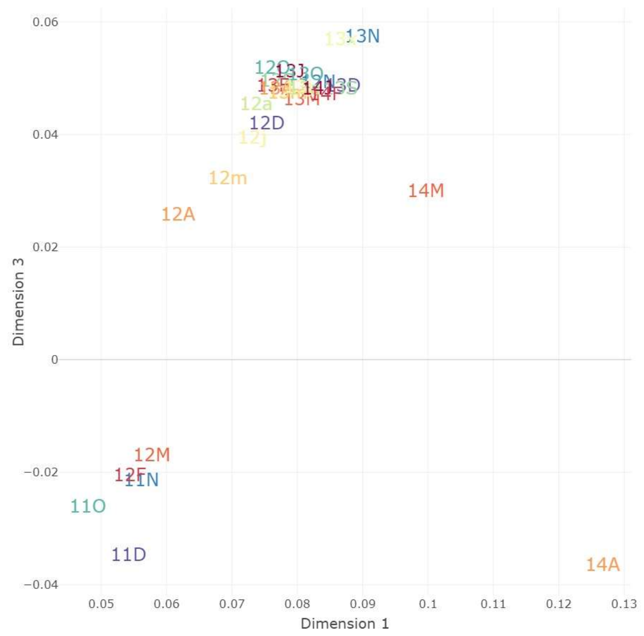
Dimension 3
Dimension 1
13N
12O
12a
12D
12j
12m
14M
12A
12M
12F
11N
11O
11D
14A

(d)

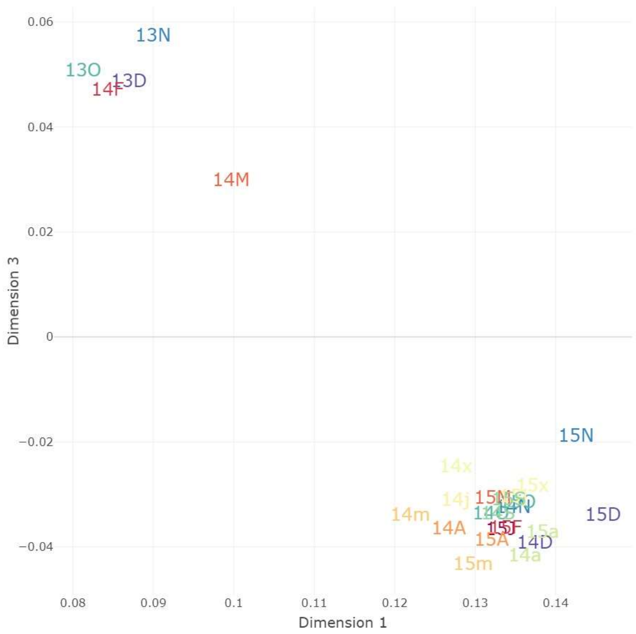
Dimension 3
Dimension 1
0.06
0.04
0.02
0
−0.02
−0.04
0.08
0.09
0.1
0.11
0.12
0.13
0.14
13N
13O
13D
14F
14M
15N
14x
15x
14j
15M
15D
14m
14A
15F
15a
15A
14D
14a
15m
