# Supplementary material for: Data-driven discovery of changes in clinical code usage over time: a case-study on changes in cardiovascular disease recording in two English electronic health records databases (2001–2015)
Source: BMJ Open. 2020 Feb 13;10(2):e034396. doi: 10.1136/bmjopen-2019-034396 (PMC7045100; doi:10.1136/bmjopen-2019-034396)
Supplement: Author's manuscript [file bmjopen-2019-034396.draft_revisions.pdf]

BMJ Open

BMJ Open is committed to open peer review. As part of this commitment we make the peer review history of every article we publish publicly available.

When an article is published we post the peer reviewers' comments and the authors' responses online. We also post the versions of the paper that were used during peer review. These are the versions that the peer review comments apply to.

The versions of the paper that follow are the versions that were submitted during the peer review process. They are not the versions of record or the final published versions. They should not be cited or distributed as the published version of this manuscript.

BMJ Open is an open access journal and the full, final, typeset and author-corrected version of record of the manuscript is available on our site with no access controls, subscription charges or pay-per-view fees (http://bmjopen.bmj.com).

If you have any questions on BMJ Open's open peer review process please email info.bmjopen@bmj.com

BMJ Open

Data-driven discovery of changes in clinical code usage over time: a case-study on changes in cardiovascular disease recording in two English electronic health records databases (2001-2015)

Journal:	*BMJ Open*
Manuscript ID	bmjopen-2019-034396
Article Type:	Original research
Date Submitted by the Author:	18-Sep-2019
Complete List of Authors:	Rockenschaub, Patrick; University College London, Institute of Health Informatics; Health Data Research UK<br>Nguyen, Vincent; University College London, Institute of Health Informatics; Health Data Research UK<br>Aldridge, Robert; University College London, Institute of Health Informatics; Health Data Research UK<br>Acosta, Dionisio; University College London, Institute of Health Informatics; Health Data Research UK<br>García Gómez, Juan Miguel; Universitat Politècnica de València, Instituto de Aplicaciones de las Tecnologías de la Información y de las Comunicaciones Avanzadas (ITACA)<br>Sáez, Carlos; Universitat Politècnica de València, Instituto de Aplicaciones de las Tecnologías de la Información y de las Comunicaciones Avanzadas (ITACA)
Keywords:	electronic health records, data quality, clinical coding, cardiovascular disease, STATISTICS & RESEARCH METHODS

SCHOLARONE™
Manuscripts

I, the Submitting Author has the right to grant and does grant on behalf of all authors of the Work (as defined in the below author licence), an exclusive licence and/or a non-exclusive licence for contributions from authors who are: i) UK Crown employees; ii) where BMJ has agreed a CC-BY licence shall apply, and/or iii) in accordance with the terms applicable for US Federal Government officers or employees acting as part of their official duties; on a worldwide, perpetual, irrevocable, royalty-free basis to BMJ Publishing Group Ltd ("BMJ") its licensees and where the relevant Journal is co-owned by BMJ to the co-owners of the Journal, to publish the Work in this journal and any other BMJ products and to exploit all rights, as set out in our licence.

The Submitting Author accepts and understands that any supply made under these terms is made by BMJ to the Submitting Author unless you are acting as an employee on behalf of your employer or a postgraduate student of an affiliated institution which is paying any applicable article publishing charge ("APC") for Open Access articles. Where the Submitting Author wishes to make the Work available on an Open Access basis (and intends to pay the relevant APC), the terms of reuse of such Open Access shall be governed by a Creative Commons licence – details of these licences and which Creative Commons licence will apply to this Work are set out in our licence referred to above.

Other than as permitted in any relevant BMJ Author's Self Archiving Policies, I confirm this Work has not been accepted for publication elsewhere, is not being considered for publication elsewhere and does not duplicate material already published. I confirm all authors consent to publication of this Work and authorise the granting of this licence.

For peer review only

Data-driven discovery of changes in clinical code usage over time: a case-study on changes in cardiovascular disease recording in two English electronic health records databases (2001-2015)

Patrick Rockenschaub[1,2], Vincent Nguyen[1,2], Robert W Aldridge[1,2], Dionisio Acosta[1,2], Juan M García-Gómez[3], Carlos Sáez[3]

[1] Institute of Health Informatics, University College London, 222 Euston Rd, London NW1 2DA, United Kingdom
[2] Health Data Research UK, London, United Kingdom
[3] Biomedical Data Science Lab (BDSLab), Instituto de Aplicaciones de las Tecnologías de la Información y de las Comunicaciones Avanzadas (ITACA), Universitat Politècnica de València, 46022 València, Spain

* Corresponding author; Email: patrick.rockenschaub.15@ucl.ac.uk; Tel. +44 207 679 8189

Keywords: electronic health records, data quality, clinical coding, cardiovascular disease statistics & research methods

Word count: 3,653

For peer review only

Abstract

Objectives
To demonstrate how data-driven variability methods can be used to identify changes in disease recording in two English electronic health records databases between 2001-2015.

Design
Repeated cross-sectional analysis that applied data-driven temporal variability methods to assess month-by-month changes in routinely-collected medical data. A measure of difference between months was calculated based on joint distributions of age, gender, socio-economic status and recorded cardiovascular diseases. Distances between months were used to identify temporal trends in data recording.

Setting
400 English primary care practices from the Clinical Practice Research Datalink (CPRD GOLD) and 451 hospital trusts from the Hospital Episode Statistics (HES).

Main outcomes
The proportion of patients (CPRD GOLD) and hospital admissions (HES) with a recorded cardiovascular disease (CPRD GOLD: coronary heart disease, heart failure, peripheral arterial disease, stroke; HES: International Classification of Disease ICD codes I20-I69/G45).

Results
Both databases showed gradual changes in cardiovascular disease recording between 2001 and 2008. The recorded prevalence of included cardiovascular diseases in CPRD GOLD increased by 47%-62%, which partially reversed after 2008. For hospital records in HES, there was a relative decrease in angina pectoris (-34.4%) and unspecified stroke (-42.3%) over the same time period, with a concomitant increase in chronic coronary heart disease (+14.3%). Multiple abrupt changes in the use of myocardial infarction codes in hospital were found in March/April 2010, 2012 and 2014, possibly linked to updates of clinical coding guidelines.

Conclusions
Identified temporal variability could be related to potentially non-medical causes such as updated coding guidelines. These artificial changes may introduce temporal correlation among diagnoses inferred from routine data, violating the assumptions of frequently used statistical methods. Temporal variability measures provided an objective and robust technique to identify, and subsequently account for, those changes in electronic health records studies without any prior knowledge of the data collection process.

For peer review only

Strengths and limitations of this study

- We are able to show previously unreported changes in coding of cardiovascular disease in two of the largest electronic health record databases in the United Kingdom.
- Temporal variability methods supports the semi-automatic identification of time trends in data recording within large and complex electronic health record databases.
- The methods can be applied to multitype and multimodal data, are robust to large sample sizes, and can be performed simultaneously for multiple variables.
- Used metrics do not assess the correctness of codes entered by general practitioners, which require other validation techniques, but are able to signal the need for further validation.

Abbreviations

CHD	Coronary heart disease
CPRD GOLD	Clinical Practice Research Datalink GOLD
EHR	Electronic health records
HES	Hospital Episode Statistics
ICD-10	International Classification of Diseases 10th revision
IMD	Index of multiple deprivation
JSD	Jensen-Shannon distance
ONS	Office for National Statistics
PAD	Peripheral arterial disease
QOF	Quality and outcomes framework

Acronyms for months

J: January, F: February, M: March, A: April, m: May, j: June, x: July, a: August, S: September, O: October, N: November, D: December

For peer review only

Introduction

Routinely-collected electronic health records (EHR) are increasingly used for clinical research [1,2]. They often pool data from different healthcare sites over multiple years, providing a readily-available and representative national sample of clinical practice. The validity of results from observational studies heavily depends on the quality of the data [3], and researchers have become increasingly aware of the importance of adequate data quality for obtaining reliable and reproducible findings [4,5]. Systematic approaches to ascertain data quality in health data repositories have traditionally focused on the data quality dimensions of completeness, correctness, and concordance [6]. For example, validation studies of English EHR databases commonly focused on whether all relevant information on the patient is recorded (completeness), to which degree the recorded information reflects reality (correctness), and whether the recorded information agrees with information in a reference dataset (concordance) [7]. While answering these questions is of vital importance, they are not the only potential sources of bias.

Other factors that influence the reuse of data are less obvious and have often been neglected despite their potentially large impact on study results. Changes in clinical procedures over time, differences in processes between healthcare sites and the introduction of new guidelines can all cause unwarranted variations in the way data items are recorded, leading to artificial trends, irregularities and breaks in the data distributions. We have previously argued that these types of data variability over time and between participating healthcare sites pose a considerable threat to the validity and reproducibility of EHR studies [8]. Studies that ignore these variations are susceptible to obtain results of limited applicability. For example, financial incentives to improve diabetes care might exaggerate increases in recorded and reported type II diabetes [9]. At worst, variability in how data is collected might even introduce spurious relationships, such as when the above mentioned improved coding of type II diabetes reduces the incidence of patients with wrongly classified type I diabetes [10].

In this study, we set out to demonstrate how data-driven methods can be used to identify irregularities in coding for clinical diagnoses over time using a recently developed, scalable approach that allows for easy comparison of similarities and differences in the distribution of demographic patient characteristics and cardiovascular diagnosis codes [8,11]. Using this method, we show how changes in coding guidelines can and have affected cardiovascular disease recording in two major English EHR databases in primary care (Clinical Practice Research Datalink - CPRD GOLD [12]) and secondary care (Hospital Episode Statistics - HES [13]) and discuss potential causes of detected variations in coding over time.

Methods

Data Sources

Clinical Practice Research Datalink (CPRD GOLD)

CPRD GOLD is a database of retrospective health records obtained directly from the practice management software (Vision, InPractice Systems LTD) of 674 primary care practices across the UK [12]. Recorded information include patient's demography, clinical symptoms, investigations, diagnoses, and tests entered by the clinician. All clinical information is coded using Read Codes, the clinical terminology used in UK primary care [14]. As of 2015, CPRD GOLD collected data from 674 practices, including data on 4.4 million actively contributing patients and 6.9 million historic patient records [12]. Primary care data used in this study was taken from the subset of 400 English practices that had existing linkage to census and hospital data. All data was obtained via the CALIBER research resource [15].

For peer review only

Hospital Episode Statistics (HES)

Hospital Episode Statistics (HES) Admitted Patient Care (APC) is a repository of hospital activity data collected as part of management, planning and reimbursement of NHS hospitals in England [13]. Information is organised in finished consultant episodes (i.e. the time spent under the uninterrupted care of a single consultant) and includes the patient's demography, admission and discharge dates, hospital diagnoses, and performed procedures. Each episode has an assigned primary diagnosis, which denotes the main condition treated during that episode, and up to 19 secondary diagnoses that contain any comorbidities relevant to the episode. Diagnoses are coded using the International Classification of Diseases 10th revision (ICD-10) codes and surgical procedures are recorded using OPCS-4 codes. In the financial year 2014/15, a total of 18.7 million episodes from 451 NHS hospital trusts were captured in HES, which was equal to 34.3 episodes per 100 person-years [13]. Data from HES used in this study was pre-aggregated by month, age group, gender, socio-economic status and 3-character ICD-10 code.

Study design and population

Using the above datasets, we conducted two cross-sectional analyses of electronic health records from English primary care (CPRD GOLD) and secondary care (HES) between 2001 and 2015. The data from each database was divided into monthly cross-sectional slices (Figure 1 - A).

For CPRD GOLD, patients contributed to any given month if they were between 20-110 years old at the beginning of the month and had been registered with their GP for at least one year. Data for each month included age (20-39, 40-59, 60-79, ≥80), gender, socio-economic status (quintiles of the patient's Index of Multiple Deprivation - IMD 2015 [16]), and presence of coronary heart disease (CHD), heart failure, peripheral arterial disease (PAD), and stroke. Patients were required to have a complete record on age, gender and socio-economic status, excluding those that did not (<0.1% of patients). Patients and practices were further required to fulfill standard data quality checks as performed and reported by CPRD [12]. All cardiovascular conditions were separately ascertained at the first day of each month. Presence of cardiovascular disease was defined as the presence of a relevant diagnosis code at any time before that date. Included diagnosis codes were directly taken from the Quality and Outcomes Framework (QOF; version 36.0), a financial incentive scheme introduced in 2004 aimed at improving the management and recording of chronic disease in primary care (see Supplementary Table 1). An absence of a diagnosis code in a patient's medical history was interpreted as absence of the disease.

For HES, allrecorded diagnosis of ICD chapters I20-I69 (including CHD, heart failure, stroke, but not PAD; Supplementary Table 2) and G45 (transient ischaemic attacks) associated with an admission of patients aged 40 years or more were counted by month and stratified by age (40-44, 45-49, 50-54, 55-59, 60-64, 65-69, 70-74, ≥75), gender and socioeconomic status (quintiles of the patient's IMD 2015). We did not distinguish between primary and secondary diagnoses and included all codes recorded during an admission. A summary of the included data for each dataset can be found in Supplementary Tables 3-6.

Temporal variability metrics

Variation in coding across months in both datasets was assessed via temporal variability methods previously proposed and tested by two of the authors [8,17]. These metrics compare changes in the frequency of a single trait (e.g. heart failure) or the joint occurrence of multiple traits (e.g. male and heart failure) within a patient population over time using the monthly joint distribution of all variables of interest. In the simplest case where all variables are categorical, the joint distribution is simply the histogram of all possible value combinations (Figure 1 - A). Temporal variability quantifies the differences in those monthly distributions based on pairwise distances between them (Figure 1 - B). For the purpose of

For peer review only

this study we used the Jensen-Shannon distance (JSD), an information theoretic measure that estimates the degree of similarity between two probability distributions [11], where 0 means equal distributions, and 1 means no-overlap on the distributions. Notably, all pairwise distances are bound and independent of sample size. The dissimilarity matrix resulting from all pairwise comparisons can be mapped into a Euclidean space using multidimensional scaling (Figure 1 - C; [18]), yielding a plot that visualises the data's evolution over time and allows a graphical analysis of data recording trajectories, i.e. systematic patterns in the data's evolution. A detailed description of the methods can be found in Sáez et al. (2015) [17] and Sáez et al. (2016) [8]. Functions to perform the temporal variability analysis were implemented in the R package *EHRtemporalVariability* (https://github.com/hms-dbmi/EHRtemporalVariability).

Statistical analysis

Empirical probability distributions of both datasets were estimated for each month by calculating the joint histogram divided by the total number of observations in each month, i.e. the proportions of observations with each possible combination of variables. For CPRD GOLD, this represents the prevalence of cardiovascular diseases at the beginning of each calendar month, stratified by age group, gender and socio-economic status. The denominator was the number of registered patients at the beginning of the month. For HES, proportions represent the relative frequency of included 3-character cardiovascular ICD-10 codes (e.g. I21 Acute Myocardial Infarction), again stratified by demography. The denominator was the total number of included cardiovascular codes recorded for a hospital admission in that month.

For each dataset, the temporal variability was calculated jointly for all covariates in a given month as described above. The estimated variability was plotted in a 3D scatter plot and visually inspected for data recording trajectories. Plots were searched for gradual trends, abrupt changes, seasonality, distinct subgroups, and outliers [19]. Where trends, breaks or discontinuities were observed, the same analysis was performed for each variable individually in order to isolate the source of the discovered deviation.

Results

The variables extracted from CPRD GOLD showed a gradual trend from one month to the next between 2001 and 2007 (Figure 2), mostly driven by changes in prevalence of cardiovascular diseases. The pattern suggested a continuous evolution of disease prevalence compatible with social factors (e.g. ageing) or incremental improvements in diagnostic coding or in clinical procedures. Smaller deviations from this overall trend could be seen at the end of 2002 and throughout 2005. Across the 8 years, the data distribution of cardiovascular disease prevalence changed with an average magnitude of about $1.5 * 10^{-3}{}_{JSD/month}$, which can be roughly viewed as a ~0.15% difference between consecutive histograms of disease prevalence. This is comparable to a monthly increase in the prevalence of a single disease from 1% to 2.2% or from 50% to 55% (the magnitude of the estimated JSD depends on the base prevalence). Changes across this period were mainly attributable to an increase in the number of patients with heart failure (from 6.7/1000 patients at the start of 2001 to 10.8/1000 by the end of 2007; +62%), stroke (from 14.4/1000 to 23.4/1000; +62%), PAD (from 7.0/1000 to 10.3/1000; +47%), and to a lesser extent CHD (from 44.7/1000 to 48.2/1000; +7.8%).

From 2008 onwards the trend shifted direction, owing to a reduction and partial reversal in the prevalence of heart failure, PAD, and most notably CHD (from 49/1000 at the beginning of 2008 to 39/1000 at the end of 2015; 20% reduction). The estimated average magnitude of change increased to $2.4 * 10^{-3}{}_{JSD/month}$ between 2008 and 2015. Starting in March 2011, the gradual pattern diverged from the relatively straight path seen before due to shifts in the socio-economic distribution of the patient population. This coincided with a substantial drop of contributing practices from more than 343 practices in January 2011 to 165 active practices in December 2015 (Supplementary Figure 1).

The distribution of cardiovascular diagnoses associated with HES admissions experienced a gradual change similar to that observed for CPRD GOLD until the end of 2008 (Figure 3). In this period, the use of codes generally stayed comparable and shifted only over the course of multiple years. Notable changes were seen for I20 Angina pectoris (-34.4%), I64 Unspecified stroke (-42.3%) and I25 Chronic CHD (+14.3%). After a transition period in 2009, distributions stopped to evolve gradually and started to cluster tightly by NHS financial year (April to March - Figure 3). There was a major shift in cardiovascular admission coding every two years (financial years 2010/11, 2012/13 and 2014/15). The abrupt changes were primarily due to differences in the ICD-10 codes used, while age, gender and socioeconomic status remained largely stable.

In particular, chapters I20-I25 experienced noticeable temporal breaks (Figure 4). While I21 Acute myocardial infarction declined in relative frequency starting in 2006, it increased from 8.0% of included codes in March 2012 to 10.9% in April 2012 (+36%) and remained stable thereafter. Simultaneously, the related code I22 Subsequent myocardial infarction (including reinfarction and recurrent infarction) dropped from 1.4% in March to 0.5% in April (-64%) and finally to ~0.1% after September 2012. I20 Angina pectoris decreased from 18.8% in January 2001 to 10.2% in March 2014 (-45.7%), after which it further declined by 2% points to 8.4% in April 2014 (-17.6%).

Discussion

We discovered both gradual and abrupt changes in the distribution of cardiovascular patient populations in two large English EHR databases between 2001 and 2015 using recently developed data quality measures (Table 1). The observed differences in cardiovascular disease coding might bias clinical phenotypes when applied over the entire study period, introducing correlation within time periods that violate the assumptions underlying common statistical methods (e.g. regression analysis). Temporal variability measures provided an objective and robust way to identify those changes without any prior knowledge of the data.

Many studies have used CPRD GOLD to look at the incidence [20–23], prevalence [20,21,23,24] and outcomes of cardiovascular disease [25,26]. However, none of these studies mentioned changes in the coding of diseases, and only one of these studies reported findings per year [23]. Changes in coding over time tend to be underreported in research papers, their identification limited to dedicated validation studies, which depending on the disease investigated may or may not exist. To the best of our knowledge, no patterns in the recording of included cardiovascular diseases have been recorded for CPRD yet. Previous validation studies in other chronic diseases did report changes in coding over time. Among others, improved coding has been reported for chronic obstructive pulmonary disease [27], diabetes [10], body mass index [28], and smoking status [29]. Improvements in primary care coding were primarily linked to the introduction of QOF in 2004, a payment scheme which incentivised better coding and management of chronic diseases. In line with these studies, we found alterations of the overall trend leading up to QOF (December 2002 - December 2003) and following its introduction (October 2004 - September 2005). However, these effects were small and the estimated prevalence had already been rising before 2003 and kept increasing after 2005. A study analysing the coding of diabetes further found a sharp increase of coded type 2 diabetes in 2004 that slowly started to decrease again after 2008 [9], around the same time that we observed a reversal of cardiovascular prevalence. It is unclear what prompted these changes, and whether they might be related. Our method further detected notable changes in patient population after 2011, mainly due to changes in the distribution of socio-economic status. This was likely related to a considerable reduction in the number of participating practices from around 350 to 165 at the end of the study period, potentially due to practices switching to new practice management software incompatible with CPRD GOLD [30].

For peer review only

Data on cardiovascular admissions from HES have been used alone or linked with primary care data from CPRD GOLD [24]. Again, to the best of our knowledge, no changes in cardiovascular disease coding in HES have been published previously. A validation study comparing coronary heart disease in HES (ICD-10 codes I20-I25) to data from the prospective UK Whitehall II cohort study [31] found a generally good agreement between the two data sources [32], suggesting reasonable recording quality in HES. An earlier study comparing data from HES and the Million Women Study [33] came to a similar conclusion [34]. However, these studies only included data up to 2013 and 2005 respectively, and did not compare data from different time periods. Other studies based on local audits or comparison to specific disease registries [35] have reported a notable underreporting of myocardial infarction cases in HES [21,36]. Any increases or decreases in the observed number of cardiovascular diseases could thus be due to improvements or deteriorations in coding. The gradual change in distributions observed until 2008 agreed with the trends observed in CPRD GOLD and could either relate to more/less focused coding for certain diseases or a slow shift in the characteristics of the underlying patient populations. The first major change occurred in March 2010, following a new version of NHS Digital / HSCIC Coding Clinic Guidance in February [37]. This guideline included provisions for stricter coding of I21 Acute Myocardial Infarction, requiring a different coding of myocardial infarction in subsequent trusts to avoid overcounting. The additional changes in 2012 and 2014 both happened in April, coinciding with the financial year of the National Health Service and the publishing of updates to the National Clinical Coding standards [38], making it likely that they too are the result of changes in coding practice. As these changes mostly occurred within the group I20-I25, they might not affect studies that use all of these codes, but may lead to problems if authors include only a single code from this group (e.g. I21). Related preliminary results on hospital admissions for all ICD-10 codes (not only chapter I) using a traditional interrupted time series analysis showed further, similar changes in non-cardiovascular chapters; these results will be disseminated in a further study evaluating life-style related diseases.

It is challenging to disentangle changes solely due to how diseases are recorded from other, genuine shifts in the patient population. While abrupt changes like the one observed here for myocardial infarction strongly suggest an exogenous cause such as new clinical coding guidelines, continuous, gradual changes over a long time period can be more difficult to classify. However, we believe that is important that researchers are aware of potential variations irrespective of the cause. Even in cases where changes are attributable to demographic shifts, accounting for them in the statistical analysis might still be warranted. The impact of observed changes, genuine as well as artificial, always depends on the specific research question [39]. For example, an increase in the estimated population prevalence of heart failure from 0.7% to 1.0% might not impact findings when accounting for heart failure as a covariate in a larger cohort, but might significantly alter the patient characteristics in a smaller, heart failure-only cohort. Similarly, while coding changes may greatly affect studies of incidence overtime, they might not change the results of a study looking at the effect of a risk factor on an outcome unless coding changes are biased towards certain patient populations. Insights gained from temporal variability analysis can be used to investigate and account for changes in patient cohorts across years.

Although some of the findings presented in this study could be detected with conventional methods such as traditional time series analysis of incidence rates, these methods usually require a formal definition of the time point at which changes happen. They further do not handle multivariate, multitype and multimodal data well [17] and require a separate analysis for each variable. This is particularly problematic when analysing changes in multinomial variables such as ICD-10 codes. More traditional methods might further struggle with large sample sizes, whereas the structure of the variability metrics allows for a flexible modelling and subsequent hypothesis testing via statistical process control. Temporal variability metrics together with the tools provided in our EHRtemporalVariability R package facilitate the calculus from raw data tables directly to visualization. Results can be shared on the

For peer review only - http://bmjopen.bmj.com/site/about/guidelines.xhtml

Shiny user interface (http://ehrtemporalvariability.upv.es), aiding transparency and communication.

Limitations

Results in this study are limited by the fact that the conditions chosen for inclusion represented a convenience sample based on the overlap between the two projects for which the data was originally obtained. The results shown here therefore do not constitute a systematic, in-depth validation study of cardiovascular disease recording. Indeed, the aim of this study was not to comprehensively investigate the data quality of cardiovascular coding in CPRD GOLD and HES but to show how systematic, data-driven methods for studying temporal variability can help to identify potential coding inconsistencies over time early on in a project and allow researchers to a priori adjust the analysis accordingly. We believe that routine checks of the temporal variability of study data will aid the validity and reproducibility of medical and epidemiological studies. Reporting coding variability in supplementary material can help readers judge the reliability of codelists and strengthen the conclusions. The analysis presented here was also limited to a manual inspection of the plots, as would be appropriate for interactive data quality analysis at the start of a project. The framework can easily be extended to allow for a more formal statistical process control (see Sáez et al. (2015 and 2018) [17,19] for guidance).

Despite the promising results, we must note that variability metrics are solely based on recorded data and will not detect the same data quality issues and trends identified by other, dedicated validation studies based on manual code review or GP questionnaires [7]. They are not meant to replace in-depth validation of data sources but rather complement them. Extensive validation studies are costly and are dependent on the exact codelists used during validation. Our data-driven approach might be well suited as a first step to signal the need for an extensive validation. With regards to the findings in this study, a reasonable first step in assessing their impact for a specific research study might be to perform analysis stratified by NHS financial year or observed stable periods. How results should be reported and whether the data is fit for purpose then depends on the results of thes sensitivity analyses and the exact research questions investigated.

Conclusion

We identified previously unreported variability in the frequency of cardiovascular codes in CPRD GOLD and HES between 2001 and 2015 using temporal variability measures that require minimal prior specification. In doing so, we have demonstrated the utility of application of data-driven approaches to data quality on two of the most important data resources for clinical research in the UK. We demonstrated that the methods can be implemented in an unsupervised, scalable manner, providing non-parametric visualisations of data recording trajectories to measure their variability. The results from this variability analysis enable researchers to adjust their analysis and ensure reproducible results.

For peer review only

Acknowledgements

We thank Dr Anoop Shah for his invaluable comments on the manuscript draft. This study is based on data from the Clinical Practice Research Datalink obtained under license from the UK Medicines and Healthcare products Regulatory Agency and on aggregated data from the Hospital Episode Statistics obtained via Public Health England. The data are provided by patients and collected by the NHS as part of their care and support. All rights reserved. All primary care data was accessed through CALIBER, which is a research resource consisting of linked electronic health record phenotypes, methods and tools, specialized infrastructure, and training and support led from the UCL Institute of Health Informatics.

Ethical approval

The primary care analysis reported in this study was approved by the Medicines and Healthcare products Regulatory Agency's independent scientific advisory committee (protocol 17 048). For analysis of secondary care data, the authors had only access to the aggregated data which was provided by Public Health England as part of VN's PhD, which they funded. No separate ethical approval was required.

Data sharing

CPRD and HES data cannot be directly shared by the researchers but are available directly from CPRD and NHS Digital subject to standard conditions. All statistical code is available from https://github.com/prockenschaub/CPRD_variability.

Author statement

PR, DA, JMGG and CS designed the study. PR and VN performed all statistical analyses reported in this study. PR wrote the manuscript. All authors interpreted the data and substantially reviewed the draft manuscript. All authors read and approved the final manuscript.

Funding statement

VN is funded by a Public Health England PhD Studentship. RWA is supported by a Wellcome Trust Clinical Research Career Development Fellowship (206602/Z/17/Z). JMGG and CS contributions to this work were partially supported by the MTS4up Spanish project (National Plan for Scientific and Technical Research and Innovation 2013-2016, No. DPI2016-80054-R), the CrowdHealth H2020-SC1-2016-CNECT project (No. 727560) (JMGG) and the Inadvance H2020-SC1-BHC-2018-2020 project (No. 825750). PR and DA did not receive any direct funding for this project.

Access to the Clinical Practice Research Datalink was supported by the UK Economic and Social Research Council (ES/P008321/1). Access to aggregated Hospital Episode Statistics was provided by Public Health England. This work was further supported by Health Data Research UK, which is funded by the UK Medical Research Council, Engineering and Physical Sciences Research Council, Economic and Social Research Council, Department of Health and Social Care (England), Chief Scientist Office of the Scottish Government Health and Social Care Directorates, Health and Social Care Research and Development Division (Welsh Government), Public Health Agency (Northern Ireland), British Heart Foundation and the Wellcome Trust.

Patient and public involvement

Anonymised patient data were used in this study. Patients and the public were not involved in the design or planning of the study.

For peer review only

Competing interests
None declared

For peer review only

Tables

Table 1 *- Variability in the Clinical Practice Research Datalink (CPRD) and Hospital Episode Statistics (HES) and their potential causes*

Clinical Practice Research Datalink (CPRD GOLD)

Finding	Observable cause	Possible original cause
Gradual change in the population distribution between 2001 and 2007	Increases in the prevalence of recorded cardiovascular disease	Demographic changes (e.g. ageing); incremental improvements in diagnostic coding or in clinical procedures
Shift in the direction of change in 2008	After the previous year's increase, the prevalence of CHD, heart failure, and PAD started decreasing again around the same time	No immediate reason identified
Oscillations in the data distributions after 2010	Changes in the distribution of socio-economic status in the target distribution	Selective drop-out of practices, possibly related to a switch in the practice management software

Hospital Episode Statistics (HES)

Finding	Observable cause	Possible original cause
Gradual change in the population distribution between 2001 and 2008	Increase in reported chronic CHD and atrial fibrillation; decreases in reported angina pectoris, acute myocardial infarction, heart failure, and stroke	Demographic changes (e.g. ageing); incremental improvements in diagnostic coding or in clinical procedures; selective increase of disease incidence
Shift in the direction of change in 2009	Increased coding of transient cerebral ischaemic attacks between 2009 and 2010	No immediate reason identified
Abrupt change in March 2010	Drop in acute myocardial infarction coding	Update to the HSCIC Coding Clinic Guidance in February 2010
Further abrupt changes in April 2012 and 2014	Sudden increase in acute myocardial infarction coding in 2012 with concomitant drop in subsequent myocardial infarction records; sudden further decrease in angina pectoris codes in 2014	Update to the National Clinical Coding Guidance National Clinical Coding Standards ICD-10 4th Edition

For peer review only

Figures

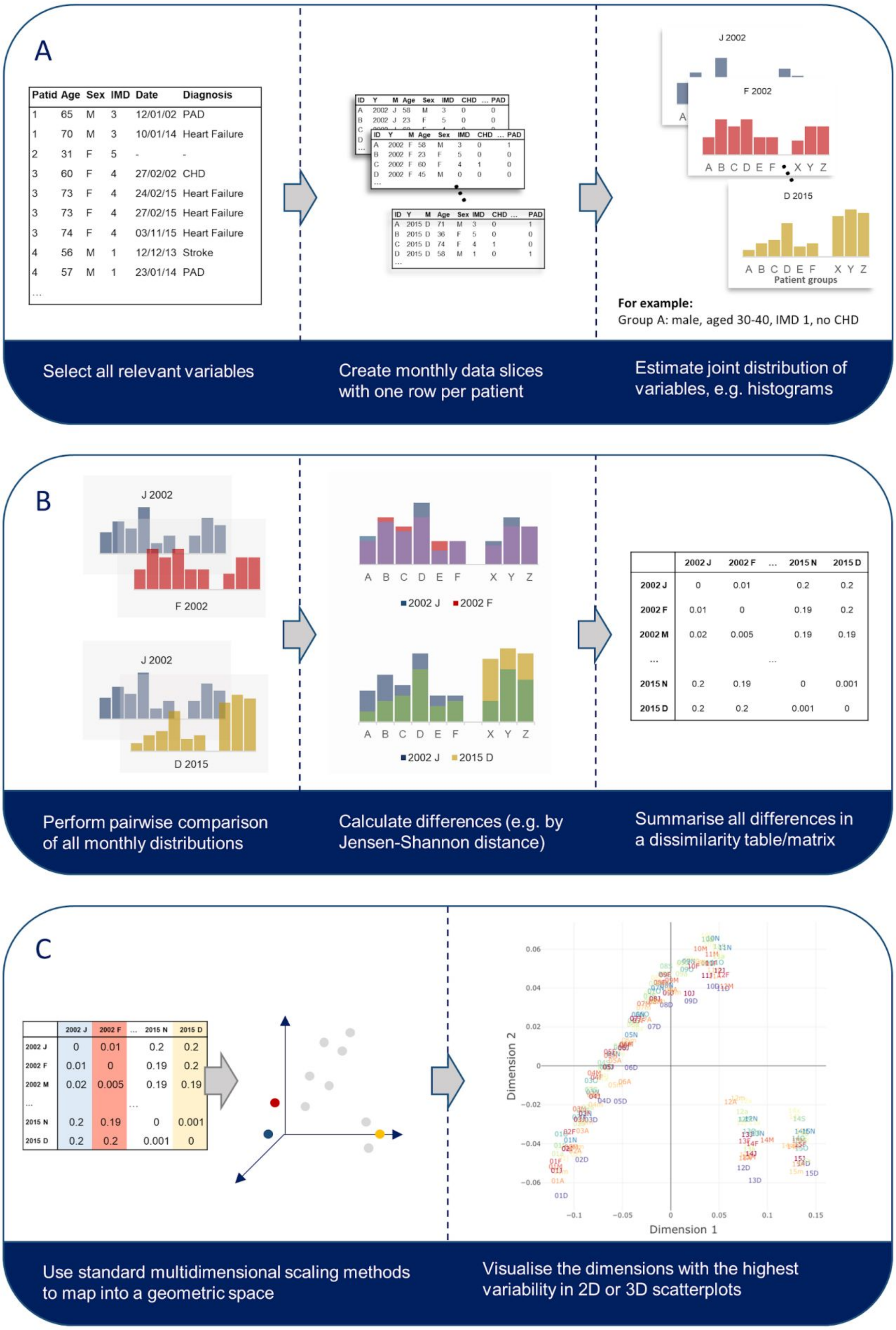

Figure 1 *- Step-by-step explanation to estimate and visualise the temporal variability of a dataset. Methods included in the R package EHRtemporalVariability were created to support researchers with all steps in this process.*

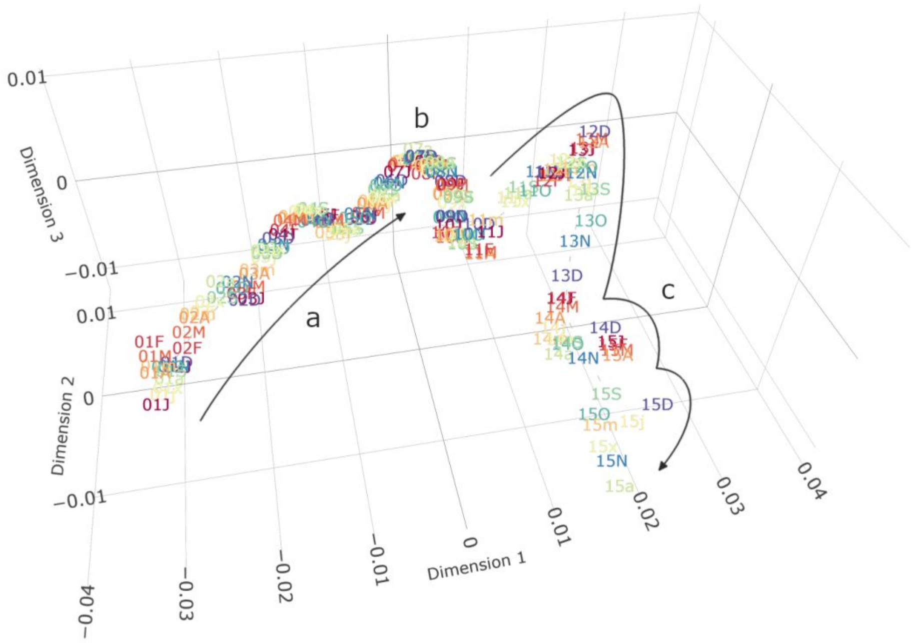

Figure 2 *- Information-Geometric Temporal (IGT) plot of demography (without age*) and cardiovascular disease prevalence in CPRD between 2001 and 2015. Each point represents joint prevalence in a single month (labelled with the last 2 digits of the year and month as given in the list of abbreviations) and distances represent the relative difference between them. Dimensions have no inherent meaning but represent the three ordered dimensions of highest variability as determined by multidimensional scaling. (a) Between 2001-2008 there was a gradual increase in disease prevalence, with two indentations corresponding to the years 2003 and 2005. (b) In 2008, the general trend reverses and prevalences decrease again, shown by a change in the direction of the graph. (c) The magnitude of variability increases after 2011, predominantly owing to changes in the socio-economic status due to a reduction in the number of practices contributing to the dataset.*

** The given graph excluded the age variable for clarity. Since CPRD GOLD includes only the year of birth, including age leads to artificial yearly jumps in July when every patient is considered one year older. The overall conclusion remains unaltered. A full graph including age can be found in the supplementary material.*

For peer review only

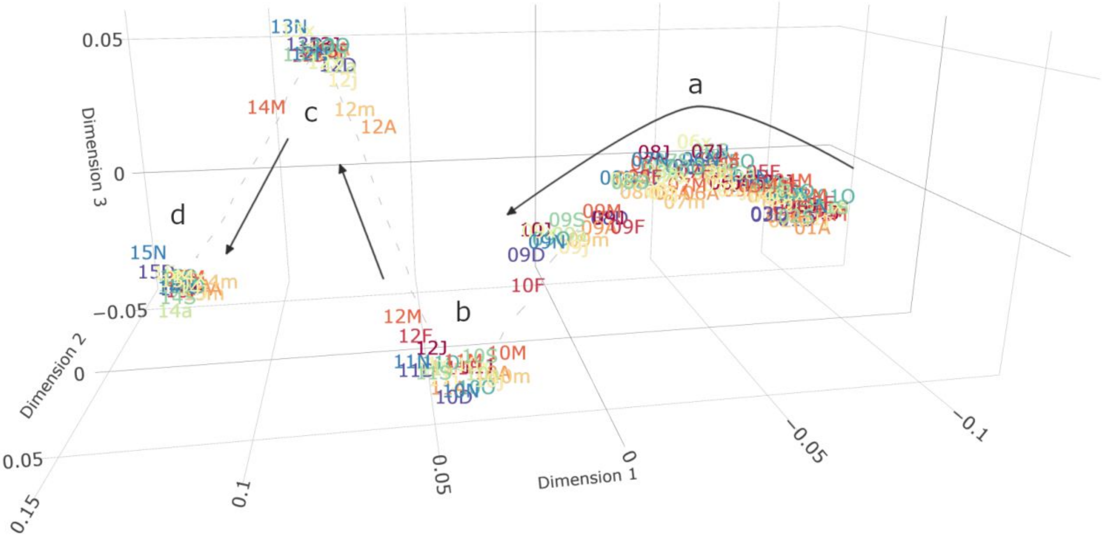

***Figure 3** - Information-Geometric Temporal (IGT) plot of demography and cardiovascular disease coding in HES between 2001 and 2015. Each point represents joint prevalence in a single month (labelled with the last 2 digits of the year and month as given in the list of abbreviations) and distances represent the relative difference between them. Dimensions have no inherent meaning but represent the three dimensions of highest variability (in order) as determined by multidimensional scaling. (a) From 2001-2009 there was a gradual change in which cardiovascular codes were associated with hospital admission. The data distributions start to diverge from the previous trend in March 2009. (b) In March 2010, the distribution of cardiovascular codes abruptly changes. (c and d) Similar and even stronger changes in cardiovascular disease coding occurred again in April 2012 and April 2014. The distributions within these 2-year batches remained stable.*

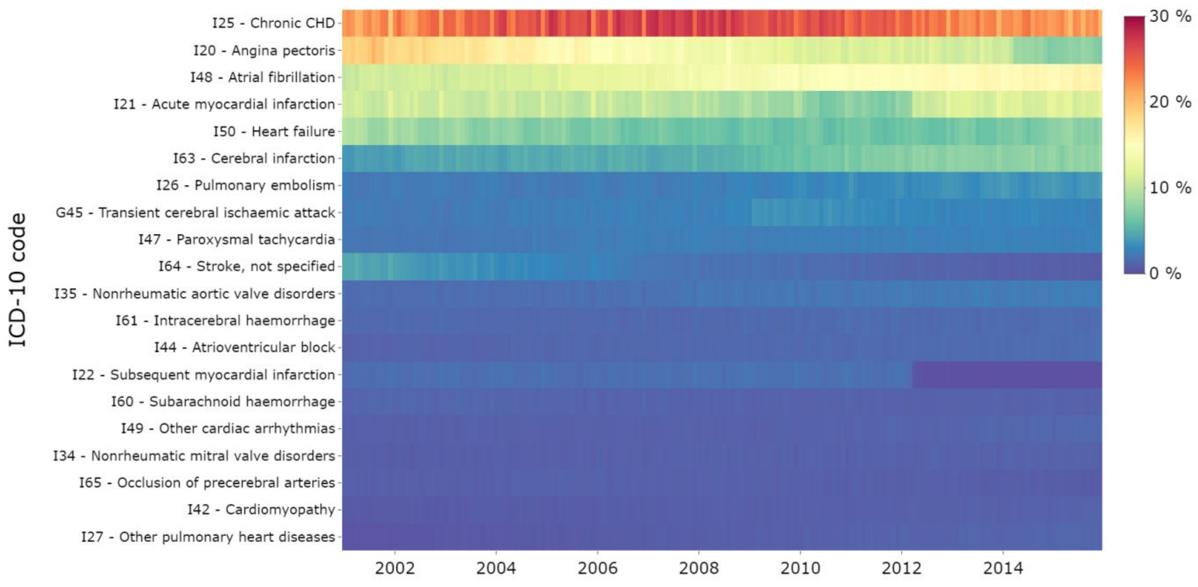

Figure 4 *- Data Temporal Map (DTM) of ICD-10 coding linked to hospital admissions in HES between 2001 and 2015. Each row represents a single ICD-10 code (3 characters) and the colour shows the proportion of admissions with that code in each month. Gradual changes in code frequency can notably be seen for I20 - Angina Pectoris, I21 - Acute Myocardial Infarction, I63 - Cerebral Infarction, and I64 - Stroke, not specified. Abrupt changes appear in the coding of G45 - Transient Cerebral ischaemic attack (2009), I21 - Acute Myocardial Infarction (2010 and 2012), and I20 - Angina Pectoris (2014).*

For peer review only

References

1 Hripcsak G, Albers DJ. Next-generation phenotyping of electronic health records. *J Am Med Inform Assoc* 2013;**20**:117–21.

2 Burton PR, Murtagh MJ, Boyd A, *et al.* Data Safe Havens in health research and healthcare. *Bioinformatics* 2015;**31**:3241–8.

3 Cruz-Correia R, Rodrigues P, Freitas A, *et al.* Data Quality and Integration Issues in Electronic Health Records. Information Discovery on Electronic Health Records. 2009. doi:10.1201/9781420090413-c4

4 Massoudi BL, Goodman KW, Gotham IJ, *et al.* An informatics agenda for public health: summarized recommendations from the 2011 AMIA PHI Conference. *J Am Med Inform Assoc* 2012;**19**:688–95.

5 Schlegel DR, Ficheur G. Secondary Use of Patient Data: Review of the Literature Published in 2016. *Yearb Med Inform* 2017;**26**:68–71.

6 Weiskopf NG, Weng C. Methods and dimensions of electronic health record data quality assessment: enabling reuse for clinical research. *J Am Med Inform Assoc* 2013;**20**:144–51.

7 Herrett E, Thomas SL, Schoonen WM, *et al.* Validation and validity of diagnoses in the General Practice Research Database: a systematic review. *Br J Clin Pharmacol* 2010;**69**:4–14.

8 Sáez C, Zurriaga O, Pérez-Panadés J, *et al.* Applying probabilistic temporal and multisite data quality control methods to a public health mortality registry in Spain: a systematic approach to quality control of repositories. *J Am Med Inform Assoc* 2016;**23**:1085–95.

9 Tate AR, Dungey S, Glew S, *et al.* Quality of recording of diabetes in the UK: how does the GP's method of coding clinical data affect incidence estimates? Cross-sectional study using the CPRD database. *BMJ Open* 2017;**7**:e012905.

10 Calvert M, Shankar A, McManus RJ, *et al.* Effect of the quality and outcomes framework on diabetes care in the United Kingdom: retrospective cohort study. *BMJ* 2009;**338**:b1870.

11 Sáez C, Robles M, García-Gómez JM. Stability metrics for multi-source biomedical data based on simplicial projections from probability distribution distances. *Stat Methods Med Res* 2017;**26**:312–36.

12 Herrett E, Gallagher AM, Bhaskaran K, *et al.* Data Resource Profile: Clinical Practice Research Datalink (CPRD). *Int J Epidemiol* 2015;**44**:827–36.

13 Herbert A, Wijlaars L, Zylbersztejn A, *et al.* Data Resource Profile: Hospital Episode Statistics Admitted Patient Care (HES APC). *Int J Epidemiol* 2017;**46**:1093–1093i.

14 Chisholm J. The Read clinical classification. *BMJ* 1990;**300**:1092.

15 Denaxas S, Gonzalez-Izquierdo A, Direk K, *et al.* UK phenomics platform for developing and validating electronic health record phenotypes: CALIBER. *J Am Med Inform Assoc* Published Online First: 22 July 2019. doi:10.1093/jamia/ocz105

16 Department for Communities and Local Government. The English Index of Multiple Deprivation (IMD) 2015 - Guidance.

For peer review only

https://assets.publishing.service.gov.uk/government/uploads/system/uploads/attachment_data/file/464430/English_Index_of_Multiple_Deprivation_2015_-_Guidance.pdf (accessed 8 Dec 2019).

17 Sáez C, Rodrigues PP, Gama J, *et al.* Probabilistic change detection and visualization methods for the assessment of temporal stability in biomedical data quality. *Data Min Knowl Discov* 2015;**29**:950–75.

18 Borg I, Groenen P. Modern Multidimensional Scaling: Theory and Applications. *J Educational Measurement* 2003;**40**:277–80.

19 Sáez C, García-Gómez JM. Kinematics of Big Biomedical Data to characterize temporal variability and seasonality of data repositories: Functional Data Analysis of data temporal evolution over non-parametric statistical manifolds. *Int J Med Inform* 2018;**119**:109–24.

20 Conrad N, Judge A, Tran J, *et al.* Temporal trends and patterns in heart failure incidence: a population-based study of 4 million individuals. *Lancet* 2018;**391**:572–80.

21 Herrett E, Shah AD, Boggon R, *et al.* Completeness and diagnostic validity of recording acute myocardial infarction events in primary care, hospital care, disease registry, and national mortality records: cohort study. *BMJ* 2013;**346**:f2350.

22 Pujades-Rodriguez M, Timmis A, Stogiannis D, *et al.* Socioeconomic deprivation and the incidence of 12 cardiovascular diseases in 1.9 million women and men: implications for risk prediction and prevention. *PLoS One* 2014;**9**:e104671.

23 Lee S, Shafe ACE, Cowie MR. UK stroke incidence, mortality and cardiovascular risk management 1999-2008: time-trend analysis from the General Practice Research Database. *BMJ Open* 2011;**1**:e000269.

24 Bhatnagar P, Wickramasinghe K, Williams J, *et al.* The epidemiology of cardiovascular disease in the UK 2014. *Heart* 2015;**101**:1182–9.

25 Taylor CJ, Ordóñez-Mena JM, Roalfe AK, *et al.* Trends in survival after a diagnosis of heart failure in the United Kingdom 2000-2017: population based cohort study. *BMJ* 2019;**364**:l223.

26 Gho JMIH, Schmidt AF, Pasea L, *et al.* An electronic health records cohort study on heart failure following myocardial infarction in England: incidence and predictors. *BMJ Open* 2018;**8**:e018331.

27 Quint JK, Müllerova H, DiSantostefano RL, *et al.* Validation of chronic obstructive pulmonary disease recording in the Clinical Practice Research Datalink (CPRD-GOLD). *BMJ Open* 2014;**4**:e005540.

28 Bhaskaran K, Forbes HJ, Douglas I, *et al.* Representativeness and optimal use of body mass index (BMI) in the UK Clinical Practice Research Datalink (CPRD). *BMJ Open* 2013;**3**:e003389.

29 Booth HP, Prevost AT, Gulliford MC. Validity of smoking prevalence estimates from primary care electronic health records compared with national population survey data for England, 2007 to 2011. *Pharmacoepidemiol Drug Saf* 2013;**22**:1357–61.

30 Booth H, Dedman D, Wolf A. CPRD Aurum Frequently asked questions (FAQs). CPRD 2019.

31 Marmot MG, Smith GD, Stansfeld S, *et al.* Health inequalities among British civil

For peer review only

servants: the Whitehall II study. *Lancet* 1991;**337**:1387–93.

32 Kivimäki M, Batty GD, Singh-Manoux A, *et al.* Validity of Cardiovascular Disease Event Ascertainment Using Linkage to UK Hospital Records. *Epidemiology* 2017;**28**:735–9.

33 Crosignani PG. Breast cancer and hormone-replacement therapy in the Million Women Study. *Maturitas* 2003;**46**:91–2.

34 Wright FL, Green J, Canoy D, *et al.* Vascular disease in women: comparison of diagnoses in hospital episode statistics and general practice records in England. *BMC Med Res Methodol* 2012;**12**:161.

35 Herrett E, Smeeth L, Walker L, *et al.* The Myocardial Ischaemia National Audit Project (MINAP). *Heart* 2010;**96**:1264–7.

36 Silver LE, Heneghan C, Mehta Z, *et al.* Substantial underestimation of incidence of acute myocardial infarction by hospital discharge diagnostic coding data: a prospective population-based study. *Heart* 2009;**95**:2–2.

37 Health and Social Care Information Centre. Coding Clinic Guidance. 5th ed. 2010.

38 Health & Social Care Information Centre. National Clinical Coding Standards - ICD-10 4th Edition. 2013. https://hscic.kahootz.com/connect.ti/t_c_home/view?objectId=31445829 (accessed 20 May 2019).

39 Wang RY, Strong DM. Beyond Accuracy: What Data Quality Means to Data Consumers. *Journal of Management Information Systems* 1996;**12**:5–33.

For peer review only

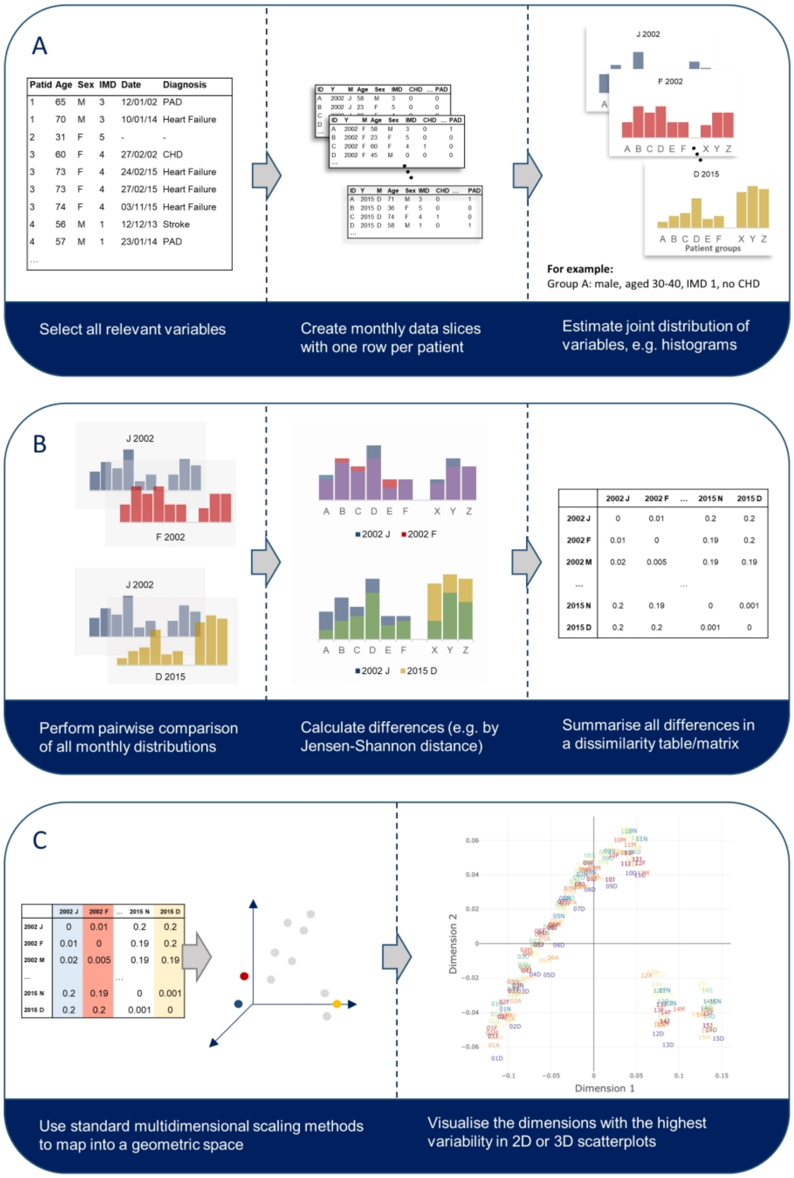

190x275mm (300 x 300 DPI)

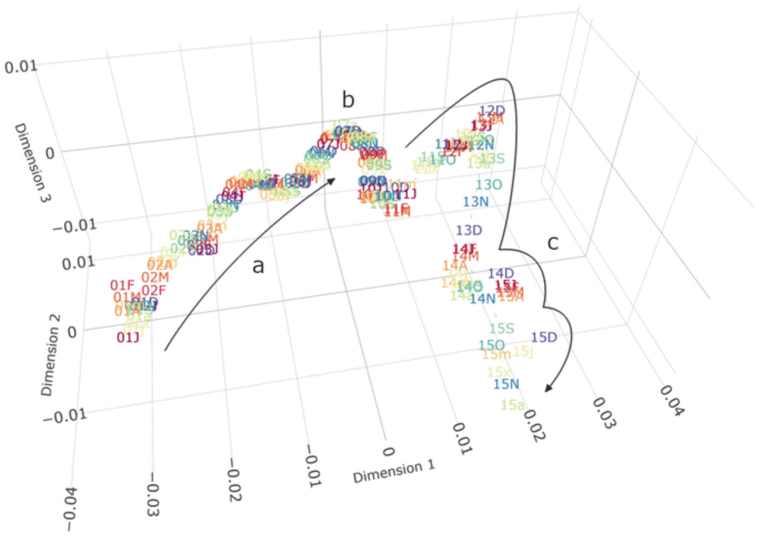

76x50mm (300 x 300 DPI)

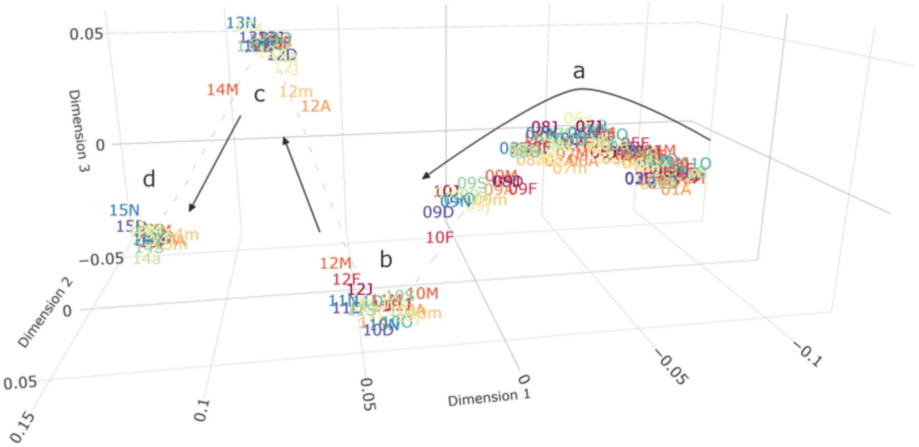

76x50mm (300 x 300 DPI)

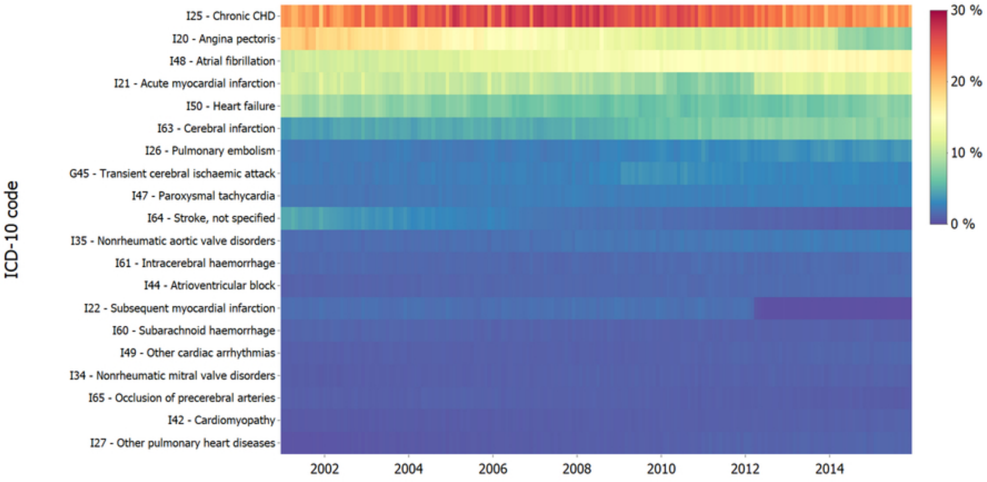

76x38mm (300 x 300 DPI)

Supplementary material

Manuscript: Data-driven discovery of changes in clinical code usage over time: a case-study on changes in cardiovascular disease recording in two English electronic health records databases (2001-2015)

Supplementary Table 1 - Read codes taken from the Quality and Outcomes Framework (QOF) used to identify history of cardiovascular disease

CHD codes	Description
G3...00	Ischaemic heart disease
G30..00	Acute myocardial infarction
G300.00	Acute anterolateral infarction
G301.00	Other specified anterior myocardial infarction
G301000	Acute anteroapical infarction
G301100	Acute anteroseptal infarction
G301z00	Anterior myocardial infarction NOS
G302.00	Acute inferolateral infarction
G303.00	Acute inferoposterior infarction
G304.00	Posterior myocardial infarction NOS
G305.00	Lateral myocardial infarction NOS
G306.00	True posterior myocardial infarction
G307.00	Acute subendocardial infarction
G307000	Acute non-Q wave infarction
G307100	Acute non-ST segment elevation myocardial infarction
G308.00	Inferior myocardial infarction NOS
G309.00	Acute Q-wave infarct
G30B.00	Acute posterolateral myocardial infarction
G30X.00	Acute transmural myocardial infarction of unspecified site
G30X000	Acute ST segment elevation myocardial infarction
G30y.00	Other acute myocardial infarction
G30y000	Acute atrial infarction
G30y100	Acute papillary muscle infarction
G30y200	Acute septal infarction
G30yz00	Other acute myocardial infarction NOS
G30z.00	Acute myocardial infarction NOS
G31..00	Other acute and subacute ischaemic heart disease
G311.00	Preinfarction syndrome
G311000	Myocardial infarction aborted

For peer review only

G311100	Unstable angina
G311200	Angina at rest
G311300	Refractory angina
G311400	Worsening angina
G311500	Acute coronary syndrome
G311z00	Preinfarction syndrome NOS
G312.00	Coronary thrombosis not resulting in myocardial infarction
G31y.00	Other acute and subacute ischaemic heart disease
G31y000	Acute coronary insufficiency
G31y100	Microinfarction of heart
G31y200	Subendocardial ischaemia
G31y300	Transient myocardial ischaemia
G31yz00	Other acute and subacute ischaemic heart disease NOS
G32..00	Old myocardial infarction
G33..00	Angina pectoris
G330.00	Angina decubitus
G330000	Nocturnal angina
G330z00	Angina decubitus NOS
G33z.00	Angina pectoris NOS
G33z000	Status anginosus
G33z100	Stenocardia
G33z200	Syncope anginosa
G33z300	Angina on effort
G33z400	Ischaemic chest pain
G33z500	Post infarct angina
G33z600	New onset angina
G33z700	Stable angina
G33zz00	Angina pectoris NOS
G34..00	Other chronic ischaemic heart disease
G340.00	Coronary atherosclerosis
G340000	Single coronary vessel disease
G340100	Double coronary vessel disease
G342.00	Atherosclerotic cardiovascular disease
G343.00	Ischaemic cardiomyopathy
G344.00	Silent myocardial ischaemia
G34y.00	Other specified chronic ischaemic heart disease
G34y000	Chronic coronary insufficiency
G34y100	Chronic myocardial ischaemia
G34yz00	Other specified chronic ischaemic heart disease NOS
G34z.00	Other chronic ischaemic heart disease NOS

For peer review only

G34z000	Asymptomatic coronary heart disease
G35..00	Subsequent myocardial infarction
G350.00	Subsequent myocardial infarction of anterior wall
G351.00	Subsequent myocardial infarction of inferior wall
G353.00	Subsequent myocardial infarction of other sites
G35X.00	Subsequent myocardial infarction of unspecified site
G38..00	Postoperative myocardial infarction
G380.00	Postoperative transmural myocardial infarction of anterior wall
G381.00	Postoperative transmural myocardial infarction of inferior wall
G382.00	Postoperative transmural myocardial infarction of other sites
G383.00	Postoperative transmural myocardial infarction of unspecified site
G384.00	Postoperative subendocardial myocardial infarction
G38z.00	Postoperative myocardial infarction, unspecified
G39..00	Coronary microvascular disease
G3y..00	Other specified ischaemic heart disease
G3z..00	Ischaemic heart disease NOS
Gyu3.00	[X]Ischaemic heart diseases
Gyu3000	[X]Other forms of angina pectoris
Gyu3200	[X]Other forms of acute ischaemic heart disease
Gyu3300	[X]Other forms of chronic ischaemic heart disease
Gyu3400	[X]Acute transmural myocardial infarction of unspecified site
Gyu3500	[X]Subsequent myocardial infarction of other sites
Gyu3600	[X]Subsequent myocardial infarction of unspecified site
**Heart failure codes**	**Description**
662f.	New York Heart Association classification - class I
662g.	New York Heart Association classification - class II
662h.	New York Heart Association classification - class III
662i.	New York Heart Association classification - class IV
G1yz1	Rheumatic left ventricular failure
G58..	Heart failure
G580.	Congestive heart failure
G5800	Acute congestive heart failure
G5801	Chronic congestive heart failure
G5802	Decompensated cardiac failure
G5803	Compensated cardiac failure
G5804	Congestive heart failure due to valvular disease
G581.	Left ventricular failure
G5810	Acute left ventricular failure
G582.	Acute heart failure

For peer review only

G583.	Heart failure with normal ejection fraction
G584.	Right ventricular failure
G58z.	Heart failure NOS
**PAD codes**	**Description**
G73..	Other peripheral vascular disease
G734.	Peripheral arterial disease
G73y.	Other specified peripheral vascular disease
G73z.	Peripheral vascular disease NOS
G73z0	Intermittent claudication
G73zz	Peripheral vascular disease NOS
Gyu74	[X]Other specified peripheral vascular diseases
**Stroke codes**	**Description**
Fyu55	[X]Other transient cerebral ischaemic attacks and related syndromes
G61..	Intracerebral haemorrhage
G610.	Cortical haemorrhage
G611.	Internal capsule haemorrhage
G612.	Basal nucleus haemorrhage
G613.	Cerebellar haemorrhage
G614.	Pontine haemorrhage
G615.	Bulbar haemorrhage
G616.	External capsule haemorrhage
G618.	Intracerebral haemorrhage, multiple localized
G619.	Lobar cerebral haemorrhage
G61X.	Intracerebral haemorrhage in hemisphere, unspecified
G61X0	Left sided intracerebral haemorrhage, unspecified
G61X1	Right sided intracerebral haemorrhage, unspecified
G61z.	Intracerebral haemorrhage NOS
G63y0	Cerebral infarct due to thrombosis of precerebral arteries
G63y1	Cerebral infarction due to embolism of precerebral arteries
G64..	Cerebral arterial occlusion
G640.	Cerebral thrombosis
G6400	Cerebral infarction due to thrombosis of cerebral arteries
G641.	Cerebral embolism
G6410	Cerebral infarction due to embolism of cerebral arteries
G64z.	Cerebral infarction NOS
G64z0	Brainstem infarction
G64z1	Wallenberg syndrome
G64z2	Left sided cerebral infarction

For peer review only

G64z3	Right sided cerebral infarction
G64z4	Infarction of basal ganglia
G65..	Transient cerebral ischaemia
G650.	Basilar artery syndrome
G651.	Vertebral artery syndrome
G6510	Vertebro-basilar artery syndrome
G652.	Subclavian steal syndrome
G653.	Carotid artery syndrome hemispheric
G654.	Multiple and bilateral precerebral artery syndromes
G656.	Vertebrobasilar insufficiency
G657.	Carotid territory transient ischaemic attack
G65y.	Other transient cerebral ischaemia
G65z.	Transient cerebral ischaemia NOS
G65z0	Impending cerebral ischaemia
G65z1	Intermittent cerebral ischaemia
G65zz	Transient cerebral ischaemia NOS
G66..	Stroke and cerebrovascular accident unspecified
G660.	Middle cerebral artery syndrome
G661.	Anterior cerebral artery syndrome
G662.	Posterior cerebral artery syndrome
G663.	Brain stem stroke syndrome
G664.	Cerebellar stroke syndrome
G665.	Pure motor lacunar syndrome
G666.	Pure sensory lacunar syndrome
G667.	Left sided CVA
G668.	Right sided CVA
G6760	Cerebral infarction due to cerebral venous thrombosis, nonpyogenic
G6W..	Cerebral infarction due to unspecified occlusion or stenosis of precerebral arteries
G6X..	Cerebral infarction due to unspecified occlusion or stenosis of cerebral arteries
Gyu62	[X]Other intracerebral haemorrhage
Gyu63	[X]Cerebral infarction due to unspecified occlusion or stenosis of cerebral arteries
Gyu64	[X]Other cerebral infarction
Gyu65	[X]Occlusion and stenosis of other precerebral arteries
Gyu66	[X]Occlusion and stenosis of other cerebral arteries
Gyu6F	[X]Intracerebral haemorrhage in hemisphere, unspecified
Gyu6G	[X]Cerebral infarction due to unspecified occlusion or stenosis of precerebral arteries
ZV12D	[V]Personal history of transient ischaemic attack

For peer review only

Supplementary Table 2 - ICD-10 chapters used to identify admissions for cardiovascular disease

ICD-10 chapter	Description
I20	Angina pectoris
I21	Acute myocardial infarction
I22	Subsequent myocardial infarction
I23	Certain current complications following acute myocardial infarction
I24	Other acute ischaemic heart diseases
I25	Chronic ischaemic heart disease
I26	Pulmonary embolism
I27	Other pulmonary heart diseases
I28	Other diseases of pulmonary vessels
I30	Acute pericarditis
I31	Other diseases of pericardium
I32	Pericarditis in diseases classified elsewhere
I33	Acute and subacute endocarditis
I34	Nonrheumatic mitral valve disorders
I35	Nonrheumatic aortic valve disorders
I36	Nonrheumatic tricuspid valve disorders
I37	Pulmonary valve disorders
I38	Endocarditis, valve unspecified
I39	Endocarditis and heart valve disorders in diseases classified elsewhere
I40	Acute myocarditis
I41	Myocarditis in diseases classified elsewhere
I42	Cardiomyopathy
I43	Cardiomyopathy in diseases classified elsewhere
I44	Atrioventricular and left bundle-branch block
I45	Other conduction disorders
I46	Cardiac arrest
I47	Paroxysmal tachycardia
I48	Atrial fibrillation and flutter
I49	Other cardiac arrhythmias
I50	Heart failure
I51	Complications and ill-defined descriptions of heart disease
I52	Other heart disorders in diseases classified elsewhere
I60	Subarachnoid haemorrhage
I61	Intracerebral haemorrhage
I62	Other nontraumatic intracranial haemorrhage
I63	Cerebral infarction
I64	Stroke, not specified as haemorrhage or infarction

For peer review only

I65	Occlusion and stenosis of precerebral arteries, not resulting in cerebral infarction
I66	Occlusion and stenosis of cerebral arteries, not resulting in cerebral infarction
I67	Other cerebrovascular diseases
I68	Cerebrovascular disorders in diseases classified elsewhere
I69	Sequelae of cerebrovascular disease

Supplementary Table 3 - Variables included in the CPRD analysis

Variable	Categories
Age	20-39, 40-59, 60-79, ≥80
Sex	Male, Female
Index of Multiple Deprivation	Quintiles
Coronary heart disease	Yes (at anytime in medical history), No
Heart failure	Yes (at anytime in medical history), No
Peripheral arterial disease	(at anytime in medical history)
Stroke	Stroke (at anytime in medical history), Transient Ischaemic Attack (at anytime in medical history but no stroke), No

Supplementary Table 4 - Univariable changes over time in CPRD (by mid-year)

	2001	2005	2010	2015
**Number of patients**	1,122,722	1,840,798	2,207,027	1,340,441
**Age (%)**				
20-39	31.6	31.2	31.9	31.5
40-59	41.1	38.9	36.8	36.8
60-79	23.8	24.2	24.5	24.8
≥80	3.4	5.7	6.7	6.9
**Female (%)**	50.9	50.6	50.6	50.8
**Index of Multiple Deprivation (mean)**	2.72	2.74	2.75	2.75
**Coronary heart disease (%)**	4.5	4.9	4.5	4.0
**Heart failure (%)**	0.7	1.0	1.0	1.0

For peer review only

**Peripheral arterial disease (%)**	0.7	1.0	1.0	0.8
**Stroke (%)**				
Transient ischaemic attack	0.7	0.9	1.0	0.9
Stroke	0.8	1.2	1.4	1.4

Note: Data on Index of Multiple Deprivation was missing for <0.1% of patients. These patients were excluded from the analysis.

Supplementary Table 5 - Variables included in the HES analysis

Variable	Categories
Age	40-44, 45-49, 50-54, 55-59, 60-64, 65-69, 70-74, ≥75
Sex	Male, Female
Index of Multiple Deprivation	Quintiles
Cardiovascular code	3-character ICD-10 code ranging from I20 to I69

Note: admissions with more than one included cardiovascular code were counted multiple times

Supplementary Table 6 - Univariable changes over time in HES (by mid-year)

	**2001**	**2005**	**2010**	**2015**
**Number of admissions**	38861	38855	39318	42756
**Age (%)**				
40-44	3.06	3.76	3.31	2.85
45-49	4.79	5.46	5.66	5.26
50-54	8.74	7.75	8.09	8.47
55-59	11.29	11.66	10.53	11.01
60-64	13.93	13.73	15.33	13.02
65-69	16.76	16.66	16.07	18.19
70-74	18.85	18.70	18.71	18.23
≥75	22.58	22.25	22.27	22.92
**Female (%)**	36.21	35.95	35.66	35.26
**Index of Multiple Deprivation (mean)**	1.74	1.79	1.77	1.76
**Cardiovascular code (top 5 %)**				

For peer review only

I25 Chronic CHD	23.4	26.52	27.39	22.94
I20 Angina pectoris	19.8	15.74	11.97	7.75
I48 Atrial fibrillation	11.29	12.72	15.56	16.85
I21 Acute myocardial infarction	10.53	10.21	6.85	11.40
I50 Heart failure	8.33	6.81	5.98	7.39

Note: There was no missing data

For peer review only

Supplementary Figure 1 - Number of primary care practice included in the analysis by month

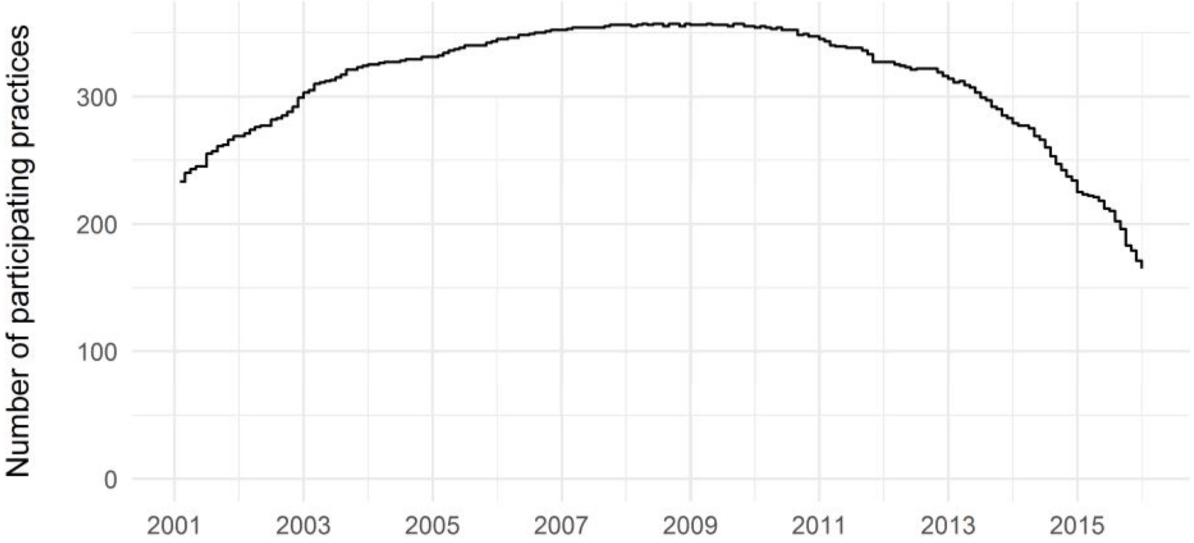

Supplementary Figure 2 - Information-Geometric Temporal (IGT) plot of demography (including age) and cardiovascular disease prevalence in CPRD between 2001 and 2015. The observed vertical columns are due to the way CPRD records age (birth year only), so changes in age can only happen in July.

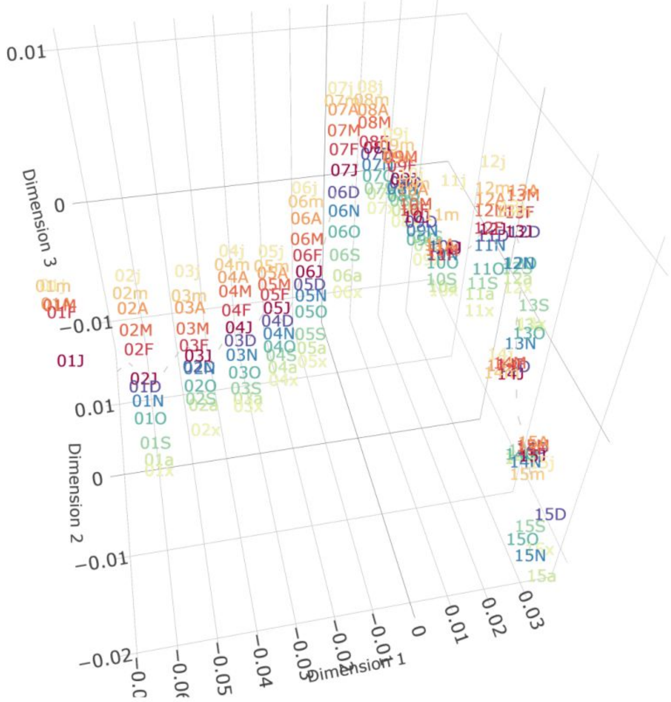

The RECORD statement – checklist of items, extended from the STROBE statement, that should be reported in observational studies using routinely collected health data.

	Item No.	STROBE items	Location in manuscript where items are reported	RECORD items	Location in manuscript where items are reported
**Title and abstract**					
	1	(a) Indicate the study's design with a commonly used term in the title or the abstract (b) Provide in the abstract an informative and balanced summary of what was done and what was found	(a)<br>This study contains a non-standard study design. It is therefore titled as a case study on EHR.<br><br>(b)<br>Abstract created in the format required by BMJ Open.	RECORD 1.1: The type of data used should be specified in the title or abstract. When possible, the name of the databases used should be included.<br><br>RECORD 1.2: If applicable, the geographic region and timeframe within which the study took place should be reported in the title or abstract.<br><br>RECORD 1.3: If linkage between databases was conducted for the study, this should be clearly stated in the title or abstract.	1.1.<br>Electronic health records databases mentioned in the title<br><br>1.2.<br>Geography and time included<br><br>1.3.<br>No linkage included.
**Introduction**					
Background rationale	2	Explain the scientific background and rationale for the investigation being reported	Introduction		
Objectives	3	State specific objectives, including any prespecified hypotheses	Introduction – paragraph		
**Methods**					
Study Design	4	Present key elements of study design early in the paper	The methods section first introduces the database, then defines the inclusion criteria, then explains the methods		

For peer review only

			used and finally discusses the specific statistical analysis.		
Setting	5	Describe the setting, locations, and relevant dates, including periods of recruitment, exposure, follow-up, and data collection	Methods – Data Sources<br>Methods – Study design and population		
Participants	6	*(a) Cohort study* - Give the eligibility criteria, and the sources and methods of selection of participants. Describe methods of follow-up<br>*Case-control study* - Give the eligibility criteria, and the sources and methods of case ascertainment and control selection. Give the rationale for the choice of cases and controls<br>*Cross-sectional study* - Give the eligibility criteria, and the sources and methods of selection of participants<br><br>*(b) Cohort study* - For matched studies, give matching criteria and number of exposed and unexposed<br>*Case-control study* - For matched studies, give matching criteria and the number of controls per case	(a)<br>Methods – Study design and population<br><br>(b)<br>No matching involved	RECORD 6.1: The methods of study population selection (such as codes or algorithms used to identify subjects) should be listed in detail. If this is not possible, an explanation should be provided.<br><br>RECORD 6.2: Any validation studies of the codes or algorithms used to select the population should be referenced. If validation was conducted for this study and not published elsewhere, detailed methods and results should be provided.<br><br>RECORD 6.3: If the study involved linkage of databases, consider use of a flow diagram or other graphical display to demonstrate the data linkage process, including the number of individuals with linked data at each stage.	6.1<br>Methods – Study design and population<br><br>Code lists separately included in the supplement<br><br>6.2<br>This is a validation study.<br><br>6.3<br>No data linkage involved.
Variables	7	Clearly define all outcomes, exposures, predictors, potential confounders, and effect modifiers. Give diagnostic criteria, if applicable.	Methods – temporal variability metrics	RECORD 7.1: A complete list of codes and algorithms used to classify exposures, outcomes, confounders, and effect modifiers should be provided. If	7.1.<br>Code lists separately included in the supplement

For peer review only

				these cannot be reported, an explanation should be provided.	
Data sources/ measurement	8	For each variable of interest, give sources of data and details of methods of assessment (measurement). Describe comparability of assessment methods if there is more than one group	Methods – data sources		
Bias	9	Describe any efforts to address potential sources of bias	This is a validation study intended to identify bias.		
Study size	10	Explain how the study size was arrived at	Study size is secondary for this analysis. Numbers and descriptive statistics are provided in the supplement.		
Quantitative variables	11	Explain how quantitative variables were handled in the analyses. If applicable, describe which groupings were chosen, and why	Methods – Study design and population		
Statistical methods	12	(a) Describe all statistical methods, including those used to control for confounding<br>(b) Describe any methods used to examine subgroups and interactions<br>(c) Explain how missing data were addressed<br>(d) *Cohort study* - If applicable, explain how loss to follow-up was addressed<br>*Case-control study* - If applicable, explain how	(a)<br>Methods – temporal variability metrics<br>Methods – statistical analysis<br><br>(b)<br>Methods – statistical analysis<br><br>(c)<br>No missing data was present		

For peer review only

		matching of cases and controls was addressed<br>*Cross-sectional study* - If applicable, describe analytical methods taking account of sampling strategy<br>(e) Describe any sensitivity analyses	(d)<br>Not applicable<br><br>(e)<br>No sensitivity analysis was performed.		
Data access and cleaning methods		..		RECORD 12.1: Authors should describe the extent to which the investigators had access to the database population used to create the study population.<br><br>RECORD 12.2: Authors should provide information on the data cleaning methods used in the study.	12.1<br>Data sharing statement<br><br>12.2<br>Code provided on Github
Linkage		..		RECORD 12.3: State whether the study included person-level, institutional-level, or other data linkage across two or more databases. The methods of linkage and methods of linkage quality evaluation should be provided.	12.3<br>No linkage involved.
**Results**					
Participants	13	(a) Report the numbers of individuals at each stage of the study (*e.g.*, numbers potentially eligible, examined for eligibility, confirmed eligible, included in the study, completing follow-up, and analysed)<br>(b) Give reasons for non-participation at each stage.<br>(c) Consider use of a flow diagram	(a)<br>Numbers provided in the supplement. Not relevant to the main argument or interpretation of the study results.<br><br>(b)<br>Inclusion was solely based on registration with the GP, as described in	RECORD 13.1: Describe in detail the selection of the persons included in the study (*i.e.,* study population selection) including filtering based on data quality, data availability and linkage. The selection of included persons can be described in the text and/or by means of the study flow diagram.	13.1<br>Methods – Study design and population<br><br>Code provided on Github

For peer review only

			Methods – Study design and population<br><br>(c)<br>Study population changes throughout the study, flow diagram not appropriate.		
Descriptive data	14	(a) Give characteristics of study participants (*e.g.*, demographic, clinical, social) and information on exposures and potential confounders<br>(b) Indicate the number of participants with missing data for each variable of interest<br>(c) *Cohort study* - summarise follow-up time (*e.g.*, average and total amount)	(a)<br>Supplementary material<br><br>(b)<br>Only socio-economic status could be missing by definition.<br>Methods – Study design and population<br><br>(c)<br>Not applicable		
Outcome data	15	*Cohort study* - Report numbers of outcome events or summary measures over time<br>*Case-control study* - Report numbers in each exposure category, or summary measures of exposure<br>*Cross-sectional study* - Report numbers of outcome events or summary measures	Not applicable – there is no outcome event		
Main results	16	(a) Give unadjusted estimates and, if applicable, confounder-	(a)<br>Not applicable		

For peer review only

		adjusted estimates and their precision (e.g., 95% confidence interval). Make clear which confounders were adjusted for and why they were included<br>(b) Report category boundaries when continuous variables were categorized<br>(c) If relevant, consider translating estimates of relative risk into absolute risk for a meaningful time period	(b)<br>Methods – Study design and population<br><br>(c)<br>Not applicable		
Other analyses	17	Report other analyses done—e.g., analyses of subgroups and interactions, and sensitivity analyses	No additional sensitivity analysis was performed		
**Discussion**					
Key results	18	Summarise key results with reference to study objectives	Discussion – paragraph 1		
Limitations	19	Discuss limitations of the study, taking into account sources of potential bias or imprecision. Discuss both direction and magnitude of any potential bias	Discussion - Limitations	RECORD 19.1: Discuss the implications of using data that were not created or collected to answer the specific research question(s). Include discussion of misclassification bias, unmeasured confounding, missing data, and changing eligibility over time, as they pertain to the study being reported.	19.1<br>This is the main topic of this analysis
Interpretation	20	Give a cautious overall interpretation of results considering objectives, limitations, multiplicity of analyses, results from similar studies, and other relevant evidence	Discussion – Limitations<br>Discussion – paragraphs 2-4		
Generalisability	21	Discuss the generalisability (external validity) of the study results	Discussion - Limitations		

Other Information					
Funding	22	Give the source of funding and the role of the funders for the present study and, if applicable, for the original study on which the present article is based	Funding statement		
Accessibility of protocol, raw data, and programming code		..		RECORD 22.1: Authors should provide information on how to access any supplemental information such as the study protocol, raw data, or programming code.	Data sharing statement<br><br>Code provided on Github

*Reference: Benchimol EI, Smeeth L, Guttmann A, Harron K, Moher D, Petersen I, Sørensen HT, von Elm E, Langan SM, the RECORD Working Committee. The REporting of studies Conducted using Observational Routinely-collected health Data (RECORD) Statement. *PLoS Medicine* 2015; in press.

*Checklist is protected under Creative Commons Attribution (CC BY) license.

For peer review only

BMJ Open

Data-driven discovery of changes in clinical code usage over time: a case-study on changes in cardiovascular disease recording in two English electronic health records databases (2001-2015)

Journal:	*BMJ Open*
Manuscript ID	bmjopen-2019-034396.R1
Article Type:	Original research
Date Submitted by the Author:	08-Jan-2020
Complete List of Authors:	Rockenschaub, Patrick; University College London, Institute of Health Informatics; Health Data Research UK<br>Nguyen, Vincent; University College London, Institute of Health Informatics; Health Data Research UK<br>Aldridge, Robert; University College London, Institute of Health Informatics; Health Data Research UK<br>Acosta, Dionisio; University College London, Institute of Health Informatics; Health Data Research UK<br>García Gómez, Juan Miguel; Universitat Politècnica de València, Instituto de Aplicaciones de las Tecnologías de la Información y de las Comunicaciones Avanzadas (ITACA)<br>Sáez, Carlos; Universitat Politècnica de València, Instituto de Aplicaciones de las Tecnologías de la Información y de las Comunicaciones Avanzadas (ITACA)
**Primary Subject Heading**:	Health informatics
Secondary Subject Heading:	Cardiovascular medicine
Keywords:	electronic health records, data quality, clinical coding, cardiovascular disease, STATISTICS & RESEARCH METHODS

SCHOLARONE™ Manuscripts

I, the Submitting Author has the right to grant and does grant on behalf of all authors of the Work (as defined in the below author licence), an exclusive licence and/or a non-exclusive licence for contributions from authors who are: i) UK Crown employees; ii) where BMJ has agreed a CC-BY licence shall apply, and/or iii) in accordance with the terms applicable for US Federal Government officers or employees acting as part of their official duties; on a worldwide, perpetual, irrevocable, royalty-free basis to BMJ Publishing Group Ltd ("BMJ") its licensees and where the relevant Journal is co-owned by BMJ to the co-owners of the Journal, to publish the Work in this journal and any other BMJ products and to exploit all rights, as set out in our licence.

The Submitting Author accepts and understands that any supply made under these terms is made by BMJ to the Submitting Author unless you are acting as an employee on behalf of your employer or a postgraduate student of an affiliated institution which is paying any applicable article publishing charge ("APC") for Open Access articles. Where the Submitting Author wishes to make the Work available on an Open Access basis (and intends to pay the relevant APC), the terms of reuse of such Open Access shall be governed by a Creative Commons licence – details of these licences and which Creative Commons licence will apply to this Work are set out in our licence referred to above.

Other than as permitted in any relevant BMJ Author's Self Archiving Policies, I confirm this Work has not been accepted for publication elsewhere, is not being considered for publication elsewhere and does not duplicate material already published. I confirm all authors consent to publication of this Work and authorise the granting of this licence.

For peer review only

Data-driven discovery of changes in clinical code usage over time: a case-study on changes in cardiovascular disease recording in two English electronic health records databases (2001-2015)

Patrick Rockenschaub[1,2], Vincent Nguyen[1,2], Robert W Aldridge[1,2], Dionisio Acosta[1,2], Juan M García-Gómez[3], Carlos Sáez[3]

[1] Institute of Health Informatics, University College London, 222 Euston Rd, London NW1 2DA, United Kingdom
[2] Health Data Research UK, London, United Kingdom
[3] Biomedical Data Science Lab (BDSLab), Instituto de Aplicaciones de las Tecnologías de la Información y de las Comunicaciones Avanzadas (ITACA), Universitat Politècnica de València, 46022 València, Spain

* Corresponding author; Email: patrick.rockenschaub.15@ucl.ac.uk; Tel. +44 207 679 8189

Keywords: electronic health records, data quality, clinical coding, cardiovascular disease statistics & research methods

Word count: 3,782

For peer review only

Abstract

Objectives

To demonstrate how data-driven variability methods can be used to identify changes in disease recording in two English electronic health records databases between 2001-2015.

Design

Repeated cross-sectional analysis that applied data-driven temporal variability methods to assess month-by-month changes in routinely-collected medical data. A measure of difference between months was calculated based on joint distributions of age, gender, socio-economic status and recorded cardiovascular diseases. Distances between months were used to identify temporal trends in data recording.

Setting

400 English primary care practices from the Clinical Practice Research Datalink (CPRD GOLD) and 451 hospital providers from the Hospital Episode Statistics (HES).

Main outcomes

The proportion of patients (CPRD GOLD) and hospital admissions (HES) with a recorded cardiovascular disease (CPRD GOLD: coronary heart disease, heart failure, peripheral arterial disease, stroke; HES: International Classification of Disease ICD codes I20-I69/G45).

Results

Both databases showed gradual changes in cardiovascular disease recording between 2001 and 2008. The recorded prevalence of included cardiovascular diseases in CPRD GOLD increased by 47%-62%, which partially reversed after 2008. For hospital records in HES, there was a relative decrease in angina pectoris (-34.4%) and unspecified stroke (-42.3%)

For peer review only

over the same time period, with a concomitant increase in chronic coronary heart disease (+14.3%). Multiple abrupt changes in the use of myocardial infarction codes in hospital were found in March/April 2010, 2012 and 2014, possibly linked to updates of clinical coding guidelines.

Conclusions

Identified temporal variability could be related to potentially non-medical causes such as updated coding guidelines. These artificial changes may introduce temporal correlation among diagnoses inferred from routine data, violating the assumptions of frequently used statistical methods. Temporal variability measures provided an objective and robust technique to identify, and subsequently account for, those changes in electronic health records studies without any prior knowledge of the data collection process.

For peer review only

Strengths and limitations of this study

- We are able to show previously unreported changes in coding of cardiovascular disease in two of the largest electronic health record databases in the United Kingdom.
- Temporal variability methods supports the semi-automatic identification of time trends in data recording within large and complex electronic health record databases.
- The methods can be applied to multitype and multimodal data, are robust to large sample sizes, and can be performed simultaneously for multiple variables.
- Used metrics do not assess the correctness of codes entered by general practitioners, which require other validation techniques, but are able to signal the need for further validation.

Abbreviations

CHD	Coronary heart disease
CPRD GOLD	Clinical Practice Research Datalink GOLD
EHR	Electronic health records
HES	Hospital Episode Statistics
ICD-10	International Classification of Diseases 10th revision
IMD	Index of multiple deprivation
JSD	Jensen-Shannon distance
ONS	Office for National Statistics
PAD	Peripheral arterial disease
QOF	Quality and outcomes framework

Acronyms for months

J: January, F: February, M: March, A: April, m: May, j: June, x: July, a: August, S: September, O: October, N: November, D: December

For peer review only

For peer review only

Introduction

Routinely-collected electronic health records (EHR) are increasingly used for clinical research [1,2]. They often pool data from different healthcare sites over multiple years, providing a readily-available and representative national sample of clinical practice. The validity of results from observational studies heavily depends on the quality of the data [3], and researchers have become increasingly aware of the importance of adequate data quality for obtaining reliable and reproducible findings [4,5]. Systematic approaches to ascertain data quality in health data repositories have traditionally focused on the data quality dimensions of completeness, correctness, and concordance [6]. For example, validation studies of English EHR databases commonly focused on whether all relevant information on the patient is recorded (completeness), to which degree the recorded information reflects reality (correctness), and whether the recorded information agrees with information in a reference dataset (concordance) [7]. While answering these questions is of vital importance, they are not the only potential sources of bias.

Other factors that influence the reuse of data are less obvious and have often been neglected despite their potentially large impact on study results. Changes in clinical procedures over time, differences in processes between healthcare sites and the introduction of new guidelines can all cause unwarranted variations in the way data items are recorded, leading to artificial trends, irregularities and breaks in the data distributions. We have previously argued that these types of data variability over time and between participating healthcare sites pose a considerable threat to the validity and reproducibility of EHR studies [8]. Studies that ignore these variations are susceptible to obtain results of limited applicability. For example, financial incentives to improve diabetes care might exaggerate increases in recorded and reported type II diabetes [9]. At worst, variability in how data is collected might even introduce spurious relationships, such as when the above mentioned improved coding of type II diabetes reduces the incidence of patients with wrongly classified type I diabetes [10].

In this study, we set out to demonstrate how data-driven methods can be used to identify irregularities in coding for clinical diagnoses over time using a recently developed, scalable approach that allows for easy comparison of similarities and differences in the distribution of demographic patient characteristics and cardiovascular diagnosis codes [8,11]. Using this method, we show how changes in coding guidelines can and have affected cardiovascular disease recording in two major English EHR databases in primary care (Clinical Practice Research Datalink - CPRD GOLD [12]) and secondary care (Hospital Episode Statistics - HES [13]) and discuss potential causes of detected variations in coding over time.

For peer review only

Methods

Data Sources

Clinical Practice Research Datalink (CPRD GOLD)

CPRD GOLD is a database of retrospective health records obtained directly from the practice management software (Vision, InPractice Systems LTD) of 674 primary care practices across the UK [12]. Recorded information include patient's demography, clinical symptoms, investigations, diagnoses, and tests entered by the clinician. All clinical information is coded using Read Codes, the clinical terminology used in UK primary care [14]. As of 2015, CPRD GOLD collected data from 674 practices, including data on 4.4 million actively contributing patients and 6.9 million historic patient records [12]. Primary care data used in this study was taken from the subset of 400 English practices that had existing linkage to census data. All data was obtained via the CALIBER research resource [15].

Hospital Episode Statistics (HES)

Hospital Episode Statistics (HES) Admitted Patient Care (APC) is a repository of hospital activity data collected as part of management, planning and reimbursement of NHS hospitals in England [13]. Information is organised in finished consultant episodes (i.e. the time spent under the uninterrupted care of a single consultant) and includes the patient's demography, admission and discharge dates, hospital diagnoses, and performed procedures. Each episode has an assigned primary diagnosis, which denotes the main condition treated during that episode, and up to 19 secondary diagnoses that contain any comorbidities relevant to the episode. Diagnoses are coded using the International Classification of Diseases 10th revision (ICD-10) codes and surgical procedures are recorded using OPCS-4 codes. In the financial year 2014/15, a total of 18.7 million episodes from 451 NHS hospital providers were captured in HES, which was equal to 34.3 episodes per 100 person-years [13]. Data from HES used in this study was pre-aggregated by month, age group, gender, socio-economic status and 3-character ICD-10 code.

Study design and population

Using the above datasets, we conducted two cross-sectional analyses of electronic health records from English primary care (CPRD GOLD) and secondary care (HES) between 2001 and 2015. The data from each database was divided into monthly cross-sectional slices (Figure 1 - A).

For CPRD GOLD, patients contributed to any given month if they were between 20-110 years old at the beginning of the month and had been registered with their GP for at least

For peer review only

one year. All patient data was ascertained on the first day of each month. Patients who left the practice during the month still contributed to that month. Data for each month included age (20-39, 40-59, 60-79, ≥80), gender, socio-economic status (quintiles of the patient's Index of Multiple Deprivation - IMD 2015 [16]), and presence of coronary heart disease (CHD), heart failure, peripheral arterial disease (PAD), and stroke. Patients were required to have a complete record on age, gender and socio-economic status, excluding those that did not (<0.1% of patients). Patients and practices were further required to fulfil standard data quality checks as performed and reported by CPRD [12]. The four cardiovascular conditions were ascertained separately and coded as present or absent. Presence of cardiovascular disease was defined as the presence of a relevant diagnosis code at any time before that month. Included diagnosis codes were directly taken from the Quality and Outcomes Framework (QOF; version 36.0), a financial incentive scheme introduced in 2004 aimed at improving the management and recording of chronic disease in primary care (see Supplementary Table 1). An absence of a diagnosis code in a patient's medical history was interpreted as absence of the disease.

For HES, all recorded diagnosis of ICD chapters I20-I69 (including CHD, heart failure, stroke, but not PAD; Supplementary Table 2) and G45 (transient ischaemic attacks) associated with an admission of patients aged 40 years or more were counted by month and stratified by age (40-44, 45-49, 50-54, 55-59, 60-64, 65-69, 70-74, ≥75), gender and socioeconomic status (quintiles of the patient's IMD 2015). The age range and classification differed from that used in CPRD due to differences in the availability of the data. We did not distinguish between primary and secondary diagnoses and included all codes recorded during an admission. A summary of the included data for each dataset can be found in Supplementary Tables 3-6.

Temporal variability metrics

Variation in coding across months in both datasets was assessed via temporal variability methods previously proposed and tested by two of the authors [8,17]. These metrics compare changes in the frequency of a single trait (e.g. heart failure) or the joint occurrence of multiple traits (e.g. male and heart failure) within a patient population over time using the monthly joint distribution of all variables of interest. In the simplest case where all variables are categorical, the joint distribution is simply the histogram of all possible value combinations (Figure 1 - A). Temporal variability quantifies the differences in those monthly distributions based on pairwise distances between them (Figure 1 - B). For the purpose of this study we used the Jensen-Shannon distance (JSD), an information theoretic measure that estimates the degree of similarity between two probability distributions [11], where 0

For peer review only

means equal distributions, and 1 means no-overlap on the distributions. Notably, all pairwise distances are bound and independent of sample size. The dissimilarity matrix resulting from all pairwise comparisons can be mapped into a Euclidean space using multidimensional scaling (Figure 1 - C; [18]), yielding a plot that visualises the data's evolution over time and allows a graphical analysis of data recording trajectories, i.e. systematic patterns in the data's evolution. A detailed description of the methods can be found in Sáez et al. (2015) [17] and Sáez et al. (2016) [8]. Functions to perform the temporal variability analysis were implemented in the R package *EHRtemporalVariability* (*https://github.com/hms-dbmi/EHRtemporalVariability*).

Statistical analysis

Empirical probability distributions of both datasets were estimated for each month by calculating the joint histogram divided by the total number of observations in each month, i.e. the proportions of observations with each possible combination of variables. For CPRD GOLD, this represents the prevalence of cardiovascular diseases at the beginning of each calendar month, stratified by age group, gender and socio-economic status. The denominator was the number of registered patients at the beginning of the month. For HES, proportions represent the relative frequency of included 3-character cardiovascular ICD-10 codes (e.g. I21 Acute Myocardial Infarction), again stratified by demography. The denominator was the total number of included cardiovascular codes recorded for a hospital admission in that month.

For each dataset, the temporal variability was calculated jointly for all covariates in a given month as described above. The estimated variability was plotted in a 3D scatter plot and visually inspected for data recording trajectories. Plots were searched for gradual trends, abrupt changes, seasonality, distinct subgroups, and outliers [19]. Where trends, breaks or discontinuities were observed, the same analysis was performed for each variable individually in order to isolate the source of the discovered deviation.

Results

The variables extracted from CPRD GOLD showed a gradual trend from one month to the next between 2001 and 2007 (Figure 2 and Supplementary Figures 1-2), mostly driven by changes in prevalence of cardiovascular diseases. The pattern suggested a continuous evolution of disease prevalence compatible with social factors (e.g. ageing) or incremental improvements in diagnostic coding or in clinical procedures. Smaller deviations from this overall trend could be seen at the end of 2002 and throughout 2005. Across the 8 years, the data distribution of cardiovascular disease prevalence changed with an average magnitude

For peer review only

of about $1.5 * 10^{-3}_{JSD/month}$, which can be roughly viewed as a ~0.15% difference between consecutive histograms of disease prevalence. For comparison, if we analysed only a single variable, a change in JSD/month of this size would be obtained if its prevalence rose from 1% to 2.2%. Note further that the estimated JSD depends on base prevalence and is larger for very small or very large prevalence. An absolutely larger, but relatively smaller, rise in prevalence from 51% of patients to 57% would also give the same JSD/month. Changes across this period were mainly attributable to an increase in the number of patients with heart failure (from 6.7/1000 patients at the start of 2001 to 10.8/1000 by the end of 2007; +62%), stroke (from 14.4/1000 to 23.4/1000; +62%), PAD (from 7.0/1000 to 10.3/1000; +47%), and to a lesser extent CHD (from 44.7/1000 to 48.2/1000; +7.8%).

From 2008 onwards the trend shifted direction, owing to a reduction and partial reversal in the prevalence of heart failure, PAD, and most notably CHD (from 49/1000 at the beginning of 2008 to 39/1000 at the end of 2015; 20% reduction). The estimated average magnitude of change increased to $2.4 * 10^{-3}_{JSD/month}$ between 2008 and 2015. Starting in March 2011, the gradual pattern diverged from the relatively straight path seen before due to shifts in the socio-economic distribution of the patient population. This coincided with a substantial drop of contributing practices from more than 343 practices in January 2011 to 165 active practices in December 2015 (Supplementary Figure 3).

The distribution of cardiovascular diagnoses associated with HES admissions experienced a gradual change similar to that observed for CPRD GOLD until the end of 2008 (Figure 3 and Supplementary Figure 4). In this period, the use of codes generally stayed comparable and shifted only over the course of multiple years. Notable changes were seen for I20 Angina pectoris (-34.4%), I64 Unspecified stroke (-42.3%) and I25 Chronic CHD (+14.3%). After a transition period in 2009, distributions stopped to evolve gradually and started to cluster tightly by NHS financial year (April to March - Figure 3). There was a major shift in cardiovascular admission coding every two years (financial years 2010/11, 2012/13 and 2014/15). The abrupt changes were primarily due to differences in the ICD-10 codes used, while age, gender and socioeconomic status remained largely stable.

In particular, chapters I20-I25 experienced noticeable temporal breaks (Figure 4). While I21 Acute myocardial infarction declined in relative frequency starting in 2006, it increased from 8.0% of included codes in March 2012 to 10.9% in April 2012 (+36%) and remained stable thereafter. Simultaneously, the related code I22 Subsequent myocardial infarction (including reinfarction and recurrent infarction) dropped from 1.4% in March to 0.5% in April (-64%) and finally to ~0.1% after September 2012. I20 Angina pectoris decreased from 18.8% in

For peer review only

January 2001 to 10.2% in March 2014 (-45.7%), after which it further declined by 2% points to 8.4% in April 2014 (-17.6%).

Discussion

We discovered both gradual and abrupt changes in the distribution of cardiovascular patient populations in two large English EHR databases between 2001 and 2015 using recently developed data quality measures (Table 1). The observed differences in cardiovascular disease coding might bias clinical phenotypes when applied over the entire study period, introducing correlation within time periods that violate the assumptions underlying common statistical methods (e.g. regression analysis). Temporal variability measures provided an objective and robust way to identify those changes without any prior knowledge of the data.

Many studies have used CPRD GOLD to look at the incidence [20–23], prevalence [20,21,23,24] and outcomes of cardiovascular disease [25,26]. However, none of these studies mentioned changes in the coding of diseases, and only one of these studies reported findings per year [23]. Changes in coding over time tend to be underreported in research papers, their identification limited to dedicated validation studies, which depending on the disease investigated may or may not exist. To the best of our knowledge, no patterns in the recording of included cardiovascular diseases have been recorded for CPRD yet. Previous validation studies in other chronic diseases did report changes in coding over time. Among others, improved coding has been reported for chronic obstructive pulmonary disease [27], diabetes [10], body mass index [28], and smoking status [29]. Improvements in primary care coding were primarily linked to the introduction of QOF in 2004, a payment scheme which incentivised better coding and management of chronic diseases. In line with these studies, we found alterations of the overall trend leading up to QOF (December 2002 - December 2003) and following its introduction (October 2004 - September 2005). However, these effects were small and the estimated prevalence had already been rising before 2003 and kept increasing after 2005. A study analysing the coding of diabetes further found a sharp increase of coded type 2 diabetes in 2004 that slowly started to decrease again after 2008 [9], around the same time that we observed a reversal of cardiovascular prevalence. It is unclear what prompted these changes, and whether they might be related. Our method further detected notable changes in patient population after 2011, mainly due to changes in the distribution of socio-economic status. This was likely related to a considerable reduction in the number of participating practices from around 350 to 165 at the end of the study period, potentially due to practices switching to new practice management software incompatible with CPRD GOLD [30].

For peer review only

Data on cardiovascular admissions from HES have been used alone or linked with primary care data from CPRD GOLD [24]. Again, to the best of our knowledge, no changes in cardiovascular disease coding in HES have been published previously. A systematic review of discharge coding accuracy reported improved accuracy over time [31]. A validation study comparing coronary heart disease in HES (ICD-10 codes I20-I25) to data from the prospective UK Whitehall II cohort study [32] found a generally good agreement between the two data sources [33], suggesting reasonable recording quality in HES. An earlier study comparing data from HES and the Million Women Study [34] came to a similar conclusion [35]. However, these studies only included data up to 2013 and 2005 respectively, and did not compare data from different time periods. Other studies based on local audits or comparison to specific disease registries [36] have reported a notable underreporting of myocardial infarction cases in HES [21,37]. Any increases or decreases in the observed number of cardiovascular diseases could thus be due to improvements or deteriorations in coding. The gradual change in distributions observed until 2008 agreed with the trends observed in CPRD GOLD and could relate to substitution of vague codes with more specific codes, better diagnostics (e.g. wider availability of CT scans), or a slow shift in the characteristics of the underlying patient populations. The first major change occurred in March 2010, following a new version of NHS Digital / HSCIC Coding Clinic Guidance in February [38]. This guideline included provisions for stricter coding of I21 Acute Myocardial Infarction, requiring a different coding of myocardial infarction in subsequent trusts to avoid overcounting. The additional changes in 2012 and 2014 both happened in April, coinciding with the financial year of the National Health Service and the publishing of updates to the National Clinical Coding standards [39], making it likely that they too are the result of changes in coding practice. As these changes mostly occurred within the group I20-I25, they might not affect studies that use all of these codes, but may lead to problems if authors include only a single code from this group (e.g. I21). Related preliminary results on hospital admissions for all ICD-10 codes (not only chapter I) using a traditional interrupted time series analysis showed further, similar changes in non-cardiovascular chapters; these results will be disseminated in a further study evaluating life-style related diseases.

It is challenging to disentangle changes solely due to how diseases are recorded from other, genuine shifts in the patient population. While abrupt changes like the one observed here for myocardial infarction strongly suggest an exogenous cause such as new clinical coding guidelines, continuous, gradual changes over a long time period can be more difficult to classify. However, we believe that is important that researchers are aware of potential variations irrespective of the cause. Even in cases where changes are attributable to demographic shifts, accounting for them in the statistical analysis might still be warranted. The impact of observed changes, genuine as well as artificial, always depends on the

For peer review only

specific research question [40]. For example, an increase in the estimated population prevalence of heart failure from 0.7% to 1.0% might not impact findings when accounting for heart failure as a covariate in a larger cohort, but might significantly alter the patient characteristics in a smaller, heart failure-only cohort. Similarly, while coding changes may greatly affect studies of incidence overtime, they might not change the results of a study looking at the effect of a risk factor on an outcome unless coding changes are biased towards certain patient populations. Insights gained from temporal variability analysis can be used to investigate and account for changes in patient cohorts across years.

Although some of the findings presented in this study could be detected with conventional methods such as traditional time series analysis of incidence rates, these methods usually require a formal definition of the time point at which changes happen. They further do not handle multivariate, multitype and multimodal data well [17] and require a separate analysis for each variable. This is particularly problematic when analysing changes in multinomial variables such as ICD-10 codes. More traditional methods might further struggle with large sample sizes, whereas the structure of the variability metrics allows for a flexible modelling and subsequent hypothesis testing via statistical process control. Temporal variability metrics together with the tools provided in our EHRtemporalVariability R package facilitate the calculus from raw data tables directly to visualization. Results can be shared on the Shiny user interface (http://ehrtemporalvariability.upv.es), aiding transparency and communication.

Limitations

Results in this study are limited by the fact that the conditions chosen for inclusion represented a convenience sample based on the overlap between the two projects for which the data was originally obtained. The results shown here therefore do not constitute a systematic, in-depth validation study of cardiovascular disease recording. Indeed, the aim of this study was not to comprehensively investigate the data quality of cardiovascular coding in CPRD GOLD and HES but to show how systematic, data-driven methods for studying temporal variability can help to identify potential coding inconsistencies over time early on in a project and allow researchers to a priori adjust the analysis accordingly. We believe that routine checks of the temporal variability of study data will aid the validity and reproducibility of medical and epidemiological studies. Reporting coding variability in supplementary material can help readers judge the reliability of codelists and strengthen the conclusions. The analysis presented here was also limited to a manual inspection of the plots, as would be appropriate for interactive data quality analysis at the start of a project. The framework can easily be extended to allow for a more formal statistical process control (see Sáez et al. (2015 and 2018) [17,19] for guidance). Finally, changes in both databases were analysed in

For peer review only

isolation. It is possible that the impact of some changes is mitigated if records from both databases are used jointly to define the presence or absence of disease [21].

Despite the promising results, we must note that variability metrics are solely based on recorded data and will not detect the same data quality issues and trends identified by other, dedicated validation studies based on manual code review or GP questionnaires [7]. They are not meant to replace in-depth validation of data sources but rather complement them. Extensive validation studies are costly and are dependent on the exact codelists used during validation. Our data-driven approach might be well suited as a first step to signal the need for an extensive validation. With regards to the findings in this study, a reasonable first step in assessing their impact for a specific research study might be to perform analysis stratified by NHS financial year or observed stable periods. How results should be reported and whether the data is fit for purpose then depends on the results of these sensitivity analyses and the exact research questions investigated.

Conclusion

We identified previously unreported variability in the frequency of cardiovascular codes in CPRD GOLD and HES between 2001 and 2015 using temporal variability measures that require minimal prior specification. In doing so, we have demonstrated the utility of application of data-driven approaches to data quality on two of the most important data resources for clinical research in the UK. We demonstrated that the methods can be implemented in an unsupervised, scalable manner, providing non-parametric visualisations of data recording trajectories to measure their variability. The results from this variability analysis enable researchers to adjust their analysis and ensure reproducible results.

For peer review only

Acknowledgements

We thank Dr Anoop Shah for his invaluable comments on reasons for the observed coding changes and the manuscript draft. This study is based on data from the Clinical Practice Research Datalink obtained under license from the UK Medicines and Healthcare products Regulatory Agency and on aggregated data from the Hospital Episode Statistics obtained via Public Health England. The data are provided by patients and collected by the NHS as part of their care and support. All rights reserved. All primary care data was accessed through CALIBER, which is a research resource consisting of linked electronic health record phenotypes, methods and tools, specialized infrastructure, and training and support led from the UCL Institute of Health Informatics.

Ethical approval

The primary care analysis reported in this study was approved by the Medicines and Healthcare products Regulatory Agency's independent scientific advisory committee [ISAC-Nr.: 17 048], under Section 251 (NHS Social Care Act 2006). For analysis of secondary care data, the authors had only access to the aggregated data which was provided by Public Health England as part of VN's PhD, which they funded. No separate ethical approval was required.

Data sharing

CPRD and HES data cannot be directly shared by the researchers but are available directly from CPRD and NHS Digital subject to standard conditions. All statistical code is available from https://github.com/prockenschaub/CPRD_variability.

Contributorship statement

PR, DA, JMGG and CS designed the study. PR and VN performed all statistical analyses reported in this study. PR wrote the manuscript. PR, VN, RWA, DA, JMGG and CS all interpreted the data and substantially reviewed the draft manuscript. All authors read and approved the final manuscript.

Funding statement

VN is funded by a Public Health England PhD Studentship. RWA is supported by a Wellcome Trust Clinical Research Career Development Fellowship (206602/Z/17/Z). JMGG and CS contributions to this work were partially supported by the MTS4up Spanish project (National Plan for Scientific and Technical Research and Innovation 2013-2016, No. DPI2016-80054-R), the CrowdHealth H2020-SC1-2016-CNECT project (No. 727560)

For peer review only

(JMGG) and the Inadvance H2020-SC1-BHC-2018-2020 project (No. 825750). PR and DA did not receive any direct funding for this project.

Access to the Clinical Practice Research Datalink was supported by the UK Economic and Social Research Council (ES/P008321/1). Access to aggregated Hospital Episode Statistics was provided by Public Health England. This work was further supported by Health Data Research UK, which is funded by the UK Medical Research Council, Engineering and Physical Sciences Research Council, Economic and Social Research Council, Department of Health and Social Care (England), Chief Scientist Office of the Scottish Government Health and Social Care Directorates, Health and Social Care Research and Development Division (Welsh Government), Public Health Agency (Northern Ireland), British Heart Foundation and the Wellcome Trust.

Patient and public involvement

Anonymised patient data were used in this study. Patients and the public were not involved in the design or planning of the study.

Competing interests

None declared

For peer review only

Tables

Table 1 *- Variability in the Clinical Practice Research Datalink (CPRD) and Hospital Episode Statistics (HES) and their potential causes and solutions*

Clinical Practice Research Datalink (CPRD GOLD)

Finding	Observable cause	Possible original cause	Possible solutions
Gradual change in the population distribution between 2001 and 2007	Increases in the prevalence of recorded cardiovascular disease	Demographic changes (e.g. ageing); incremental improvements in diagnostic coding or in clinical procedures	Incremental learning of models; inclusion of time interaction effect
Shift in the direction of change in 2008	After the previous year's increase, the prevalence of CHD, heart failure, and PAD started decreasing again around the same time	No immediate reason identified	Separate analyses of pre- and post-change data
Oscillations in the data distributions after 2010	Changes in the distribution of socio-economic status in the target distribution	Selective drop-out of practices, possibly related to a switch in the practice management software	Mixed models with practice effects

Hospital Episode Statistics (HES)

Finding	Observable cause	Possible original cause	Possible solutions
Gradual change in the population distribution between 2001 and 2008	Increase in reported chronic CHD and atrial fibrillation; decreases in reported angina pectoris, acute myocardial infarction, heart failure, and stroke	Demographic changes (e.g. ageing); incremental improvements in diagnostic coding or in clinical procedures; selective increase of disease incidence	Incremental learning of models; inclusion of continuous time interaction effect
Shift in the direction of change in 2009	Increased coding of transient cerebral ischaemic attacks between 2009 and 2010	No immediate reason identified	Separate analyses of pre- and post-change data
Abrupt change in March 2010	Drop in acute myocardial infarction coding	Update to the HSCIC Coding Clinic Guidance in February 2010	Separate analyses; incremental learning of models
Further abrupt changes in April 2012 and 2014	Sudden increase in acute myocardial infarction coding in 2012 with concomitant drop in subsequent myocardial infarction records; sudden further decrease in angina pectoris codes in 2014	Update to the National Clinical Coding Guidance National Clinical Coding Standards ICD-10 4th Edition	Separate analyses; incremental learning of models

Figure legends

Figure 1 - *Step-by-step explanation to estimate and visualise the temporal variability of a dataset. Methods included in the R package EHRtemporalVariability were created to support researchers with all steps in this process.*

Figure 2 *- Information-Geometric Temporal (IGT) plot of demography (without age*) and cardiovascular disease prevalence in CPRD between 2001 and 2015. Each point represents joint prevalence in a single month (labelled with the last 2 digits of the year and month as given in the list of abbreviations) and distances represent the relative difference between them. Dimensions have no inherent meaning but represent the three ordered dimensions of highest variability as determined by multidimensional scaling. (a) Between 2001-2008 there was a gradual increase in disease prevalence, with two indentations corresponding to the years 2003 and 2005. (b) In 2008, the general trend reverses and prevalences decrease again, shown by a change in the direction of the graph. (c) The magnitude of variability increases after 2011, predominantly owing to changes in the socio-economic status due to a reduction in the number of practices contributing to the dataset. Detailed subplots of a, b and c can be found in the supplementary material (Supplementary Figure 1).*

** The given graph excluded the age variable for clarity. Since CPRD GOLD includes only the year of birth, including age leads to artificial yearly jumps in July when every patient is considered one year older. The overall conclusion remains unaltered. A full graph including age can be found in the supplementary material (Supplementary Figure 2).*

Figure 3 *- Information-Geometric Temporal (IGT) plot of demography and cardiovascular disease coding in HES between 2001 and 2015. Each point represents joint prevalence in a single month (labelled with the last 2 digits of the year and month as given in the list of abbreviations) and distances represent the relative difference between them. Dimensions have no inherent meaning but represent the three dimensions of highest variability (in order) as determined by multidimensional scaling. (a) From 2001-2009 there was a gradual change in which cardiovascular codes were associated with hospital admission. The data distributions starts to diverge from the previous trend in March 2009. (b) In March 2010, the distribution of cardiovascular codes abruptly changes. (c and d) Similar and even stronger changes in cardiovascular disease coding occurred again in April 2012 and April 2014. The distributions within these 2-year batches remained stable. Detailed subplots of a, b, c and d can be found in the supplementary material (Supplementary Figure 4).*

Figure 4 *- Data Temporal Map (DTM) of ICD-10 coding linked to hospital admissions in HES between 2001 and 2015. Each row represents a single ICD-10 code (3 characters) and the colour shows the proportion of admissions with that code in each month. Gradual changes in code frequency can notably be seen for I20 - Angina Pectoris, I21 - Acute Myocardial Infarction, I63 - Cerebral Infarction, and I64 - Stroke, not specified. Abrupt changes appear in the coding of G45 - Transient Cerebral ischaemic attack (2009), I21 - Acute Myocardial Infarction (2010 and 2012), and I20 - Angina Pectoris (2014).*

For peer review only

References

1 Hripcsak G, Albers DJ. Next-generation phenotyping of electronic health records. *J Am Med Inform Assoc* 2013;**20**:117–21.

2 Burton PR, Murtagh MJ, Boyd A, *et al.* Data Safe Havens in health research and healthcare. *Bioinformatics* 2015;**31**:3241–8.

3 Cruz-Correia R, Rodrigues P, Freitas A, *et al.* Data Quality and Integration Issues in Electronic Health Records. Information Discovery on Electronic Health Records. 2009. doi:10.1201/9781420090413-c4

4 Massoudi BL, Goodman KW, Gotham IJ, *et al.* An informatics agenda for public health: summarized recommendations from the 2011 AMIA PHI Conference. *J Am Med Inform Assoc* 2012;**19**:688–95.

5 Schlegel DR, Ficheur G. Secondary Use of Patient Data: Review of the Literature Published in 2016. *Yearb Med Inform* 2017;**26**:68–71.

6 Weiskopf NG, Weng C. Methods and dimensions of electronic health record data quality assessment: enabling reuse for clinical research. *J Am Med Inform Assoc* 2013;**20**:144–51.

7 Herrett E, Thomas SL, Schoonen WM, *et al.* Validation and validity of diagnoses in the General Practice Research Database: a systematic review. *Br J Clin Pharmacol* 2010;**69**:4–14.

8 Sáez C, Zurriaga O, Pérez-Panadés J, *et al.* Applying probabilistic temporal and multisite data quality control methods to a public health mortality registry in Spain: a systematic approach to quality control of repositories. *J Am Med Inform Assoc* 2016;**23**:1085–95.

9 Tate AR, Dungey S, Glew S, *et al.* Quality of recording of diabetes in the UK: how does the GP's method of coding clinical data affect incidence estimates? Cross-sectional study using the CPRD database. *BMJ Open* 2017;**7**:e012905.

10 Calvert M, Shankar A, McManus RJ, *et al.* Effect of the quality and outcomes framework on diabetes care in the United Kingdom: retrospective cohort study. *BMJ* 2009;**338**:b1870.

11 Sáez C, Robles M, García-Gómez JM. Stability metrics for multi-source biomedical data based on simplicial projections from probability distribution distances. *Stat Methods Med Res* 2017;**26**:312–36.

12 Herrett E, Gallagher AM, Bhaskaran K, *et al.* Data Resource Profile: Clinical Practice Research Datalink (CPRD). *Int J Epidemiol* 2015;**44**:827–36.

13 Herbert A, Wijlaars L, Zylbersztejn A, *et al.* Data Resource Profile: Hospital Episode Statistics Admitted Patient Care (HES APC). *Int J Epidemiol* 2017;**46**:1093–1093i.

14 Chisholm J. The Read clinical classification. *BMJ* 1990;**300**:1092.

15 Denaxas S, Gonzalez-Izquierdo A, Direk K, *et al.* UK phenomics platform for developing and validating electronic health record phenotypes: CALIBER. *J Am Med Inform Assoc* Published Online First: 22 July 2019. doi:10.1093/jamia/ocz105

16 Department for Communities and Local Government. The English Index of Multiple Deprivation (IMD) 2015 - Guidance.

For peer review only

https://assets.publishing.service.gov.uk/government/uploads/system/uploads/attachment_data/file/464430/English_Index_of_Multiple_Deprivation_2015_-_Guidance.pdf (accessed 8 Dec 2019).

17 Sáez C, Rodrigues PP, Gama J, *et al.* Probabilistic change detection and visualization methods for the assessment of temporal stability in biomedical data quality. *Data Min Knowl Discov* 2015;**29**:950–75.

18 Borg I, Groenen P. Modern Multidimensional Scaling: Theory and Applications. *J Educational Measurement* 2003;**40**:277–80.

19 Sáez C, García-Gómez JM. Kinematics of Big Biomedical Data to characterize temporal variability and seasonality of data repositories: Functional Data Analysis of data temporal evolution over non-parametric statistical manifolds. *Int J Med Inform* 2018;**119**:109–24.

20 Conrad N, Judge A, Tran J, *et al.* Temporal trends and patterns in heart failure incidence: a population-based study of 4 million individuals. *Lancet* 2018;**391**:572–80.

21 Herrett E, Shah AD, Boggon R, *et al.* Completeness and diagnostic validity of recording acute myocardial infarction events in primary care, hospital care, disease registry, and national mortality records: cohort study. *BMJ* 2013;**346**:f2350.

22 Pujades-Rodriguez M, Timmis A, Stogiannis D, *et al.* Socioeconomic deprivation and the incidence of 12 cardiovascular diseases in 1.9 million women and men: implications for risk prediction and prevention. *PLoS One* 2014;**9**:e104671.

23 Lee S, Shafe ACE, Cowie MR. UK stroke incidence, mortality and cardiovascular risk management 1999-2008: time-trend analysis from the General Practice Research Database. *BMJ Open* 2011;**1**:e000269.

24 Bhatnagar P, Wickramasinghe K, Williams J, *et al.* The epidemiology of cardiovascular disease in the UK 2014. *Heart* 2015;**101**:1182–9.

25 Taylor CJ, Ordóñez-Mena JM, Roalfe AK, *et al.* Trends in survival after a diagnosis of heart failure in the United Kingdom 2000-2017: population based cohort study. *BMJ* 2019;**364**:l223.

26 Gho JMIH, Schmidt AF, Pasea L, *et al.* An electronic health records cohort study on heart failure following myocardial infarction in England: incidence and predictors. *BMJ Open* 2018;**8**:e018331.

27 Quint JK, Müllerova H, DiSantostefano RL, *et al.* Validation of chronic obstructive pulmonary disease recording in the Clinical Practice Research Datalink (CPRD-GOLD). *BMJ Open* 2014;**4**:e005540.

28 Bhaskaran K, Forbes HJ, Douglas I, *et al.* Representativeness and optimal use of body mass index (BMI) in the UK Clinical Practice Research Datalink (CPRD). *BMJ Open* 2013;**3**:e003389.

29 Booth HP, Prevost AT, Gulliford MC. Validity of smoking prevalence estimates from primary care electronic health records compared with national population survey data for England, 2007 to 2011. *Pharmacoepidemiol Drug Saf* 2013;**22**:1357–61.

30 Booth H, Dedman D, Wolf A. CPRD Aurum Frequently asked questions (FAQs). CPRD 2019.

31 Burns EM, Rigby E, Mamidanna R, *et al.* Systematic review of discharge coding

For peer review only

accuracy. *J Public Health* 2012;**34**:138–48.

32 Marmot MG, Smith GD, Stansfeld S, *et al.* Health inequalities among British civil servants: the Whitehall II study. *Lancet* 1991;**337**:1387–93.

33 Kivimäki M, Batty GD, Singh-Manoux A, *et al.* Validity of Cardiovascular Disease Event Ascertainment Using Linkage to UK Hospital Records. *Epidemiology* 2017;**28**:735–9.

34 Crosignani PG. Breast cancer and hormone-replacement therapy in the Million Women Study. *Maturitas* 2003;**46**:91–2.

35 Wright FL, Green J, Canoy D, *et al.* Vascular disease in women: comparison of diagnoses in hospital episode statistics and general practice records in England. *BMC Med Res Methodol* 2012;**12**:161.

36 Herrett E, Smeeth L, Walker L, *et al.* The Myocardial Ischaemia National Audit Project (MINAP). *Heart* 2010;**96**:1264–7.

37 Silver LE, Heneghan C, Mehta Z, *et al.* Substantial underestimation of incidence of acute myocardial infarction by hospital discharge diagnostic coding data: a prospective population-based study. *Heart* 2009;**95**:2–2.

38 Health and Social Care Information Centre. Coding Clinic Guidance. 5th ed. 2010.

39 Health & Social Care Information Centre. National Clinical Coding Standards - ICD-10 4th Edition. 2013. https://hscic.kahootz.com/connect.ti/t_c_home/view?objectId=31445829 (accessed 20 May 2019).

40 Wang RY, Strong DM. Beyond Accuracy: What Data Quality Means to Data Consumers. *Journal of Management Information Systems* 1996;**12**:5–33.

For peer review only

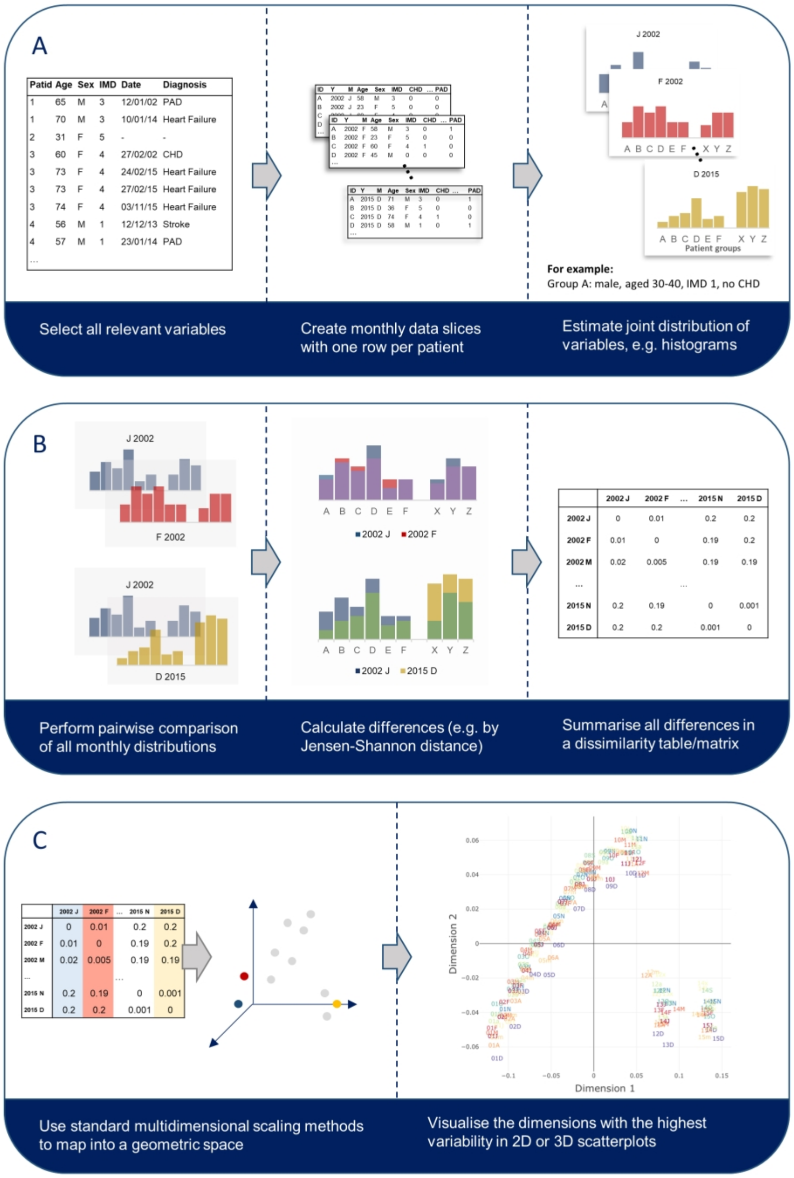

190x275mm (300 x 300 DPI)

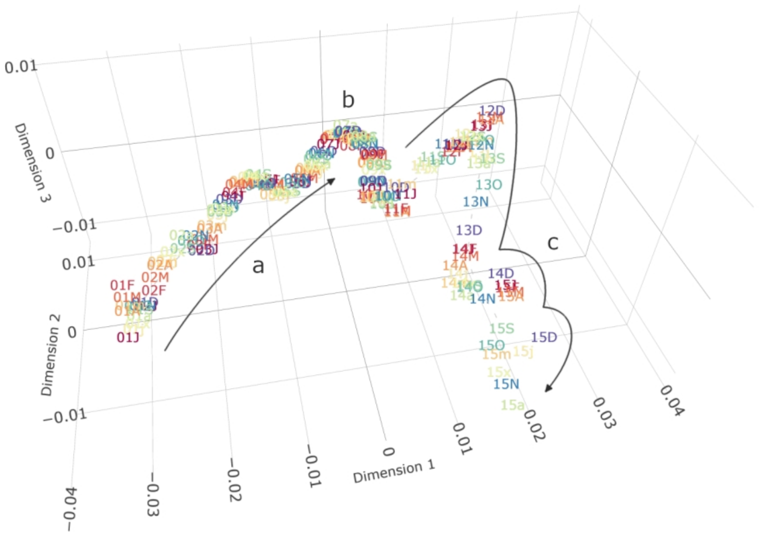

76x50mm (600 x 600 DPI)

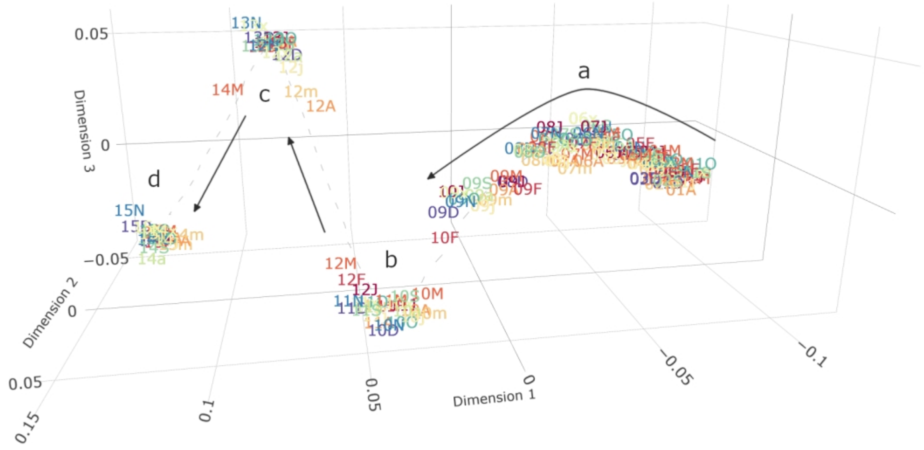

76x50mm (600 x 600 DPI)

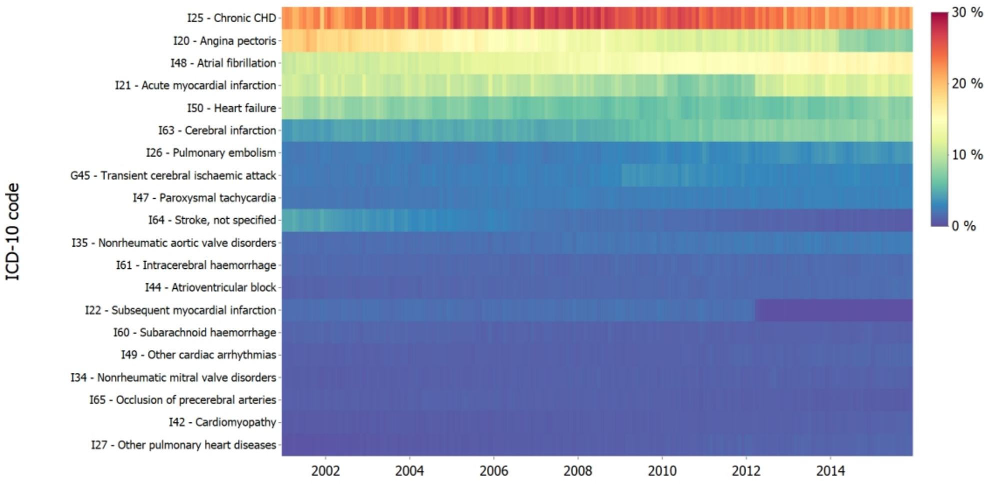

76x38mm (600 x 600 DPI)

Supplementary material

Manuscript: Data-driven discovery of changes in clinical code usage over time: a case-study on changes in cardiovascular disease recording in two English electronic health records databases (2001-2015)

Supplementary Table 1 - Read codes taken from the Quality and Outcomes Framework (QOF) used to identify history of cardiovascular disease

CHD codes	Description
G3...00	Ischaemic heart disease
G30..00	Acute myocardial infarction
G300.00	Acute anterolateral infarction
G301.00	Other specified anterior myocardial infarction
G301000	Acute anteroapical infarction
G301100	Acute anteroseptal infarction
G301z00	Anterior myocardial infarction NOS
G302.00	Acute inferolateral infarction
G303.00	Acute inferoposterior infarction
G304.00	Posterior myocardial infarction NOS
G305.00	Lateral myocardial infarction NOS
G306.00	True posterior myocardial infarction
G307.00	Acute subendocardial infarction
G307000	Acute non-Q wave infarction
G307100	Acute non-ST segment elevation myocardial infarction
G308.00	Inferior myocardial infarction NOS
G309.00	Acute Q-wave infarct
G30B.00	Acute posterolateral myocardial infarction
G30X.00	Acute transmural myocardial infarction of unspecified site
G30X000	Acute ST segment elevation myocardial infarction
G30y.00	Other acute myocardial infarction
G30y000	Acute atrial infarction
G30y100	Acute papillary muscle infarction
G30y200	Acute septal infarction
G30yz00	Other acute myocardial infarction NOS
G30z.00	Acute myocardial infarction NOS
G31..00	Other acute and subacute ischaemic heart disease
G311.00	Preinfarction syndrome
G311000	Myocardial infarction aborted

For peer review only

G311100	Unstable angina
G311200	Angina at rest
G311300	Refractory angina
G311400	Worsening angina
G311500	Acute coronary syndrome
G311z00	Preinfarction syndrome NOS
G312.00	Coronary thrombosis not resulting in myocardial infarction
G31y.00	Other acute and subacute ischaemic heart disease
G31y000	Acute coronary insufficiency
G31y100	Microinfarction of heart
G31y200	Subendocardial ischaemia
G31y300	Transient myocardial ischaemia
G31yz00	Other acute and subacute ischaemic heart disease NOS
G32..00	Old myocardial infarction
G33..00	Angina pectoris
G330.00	Angina decubitus
G330000	Nocturnal angina
G330z00	Angina decubitus NOS
G33z.00	Angina pectoris NOS
G33z000	Status anginosus
G33z100	Stenocardia
G33z200	Syncope anginosa
G33z300	Angina on effort
G33z400	Ischaemic chest pain
G33z500	Post infarct angina
G33z600	New onset angina
G33z700	Stable angina
G33zz00	Angina pectoris NOS
G34..00	Other chronic ischaemic heart disease
G340.00	Coronary atherosclerosis
G340000	Single coronary vessel disease
G340100	Double coronary vessel disease
G342.00	Atherosclerotic cardiovascular disease
G343.00	Ischaemic cardiomyopathy
G344.00	Silent myocardial ischaemia
G34y.00	Other specified chronic ischaemic heart disease
G34y000	Chronic coronary insufficiency
G34y100	Chronic myocardial ischaemia
G34yz00	Other specified chronic ischaemic heart disease NOS
G34z.00	Other chronic ischaemic heart disease NOS

For peer review only

G34z000	Asymptomatic coronary heart disease
G35..00	Subsequent myocardial infarction
G350.00	Subsequent myocardial infarction of anterior wall
G351.00	Subsequent myocardial infarction of inferior wall
G353.00	Subsequent myocardial infarction of other sites
G35X.00	Subsequent myocardial infarction of unspecified site
G38..00	Postoperative myocardial infarction
G380.00	Postoperative transmural myocardial infarction of anterior wall
G381.00	Postoperative transmural myocardial infarction of inferior wall
G382.00	Postoperative transmural myocardial infarction of other sites
G383.00	Postoperative transmural myocardial infarction of unspecified site
G384.00	Postoperative subendocardial myocardial infarction
G38z.00	Postoperative myocardial infarction, unspecified
G39..00	Coronary microvascular disease
G3y..00	Other specified ischaemic heart disease
G3z..00	Ischaemic heart disease NOS
Gyu3.00	[X]Ischaemic heart diseases
Gyu3000	[X]Other forms of angina pectoris
Gyu3200	[X]Other forms of acute ischaemic heart disease
Gyu3300	[X]Other forms of chronic ischaemic heart disease
Gyu3400	[X]Acute transmural myocardial infarction of unspecified site
Gyu3500	[X]Subsequent myocardial infarction of other sites
Gyu3600	[X]Subsequent myocardial infarction of unspecified site
**Heart failure codes**	**Description**
662f.	New York Heart Association classification - class I
662g.	New York Heart Association classification - class II
662h.	New York Heart Association classification - class III
662i.	New York Heart Association classification - class IV
G1yz1	Rheumatic left ventricular failure
G58..	Heart failure
G580.	Congestive heart failure
G5800	Acute congestive heart failure
G5801	Chronic congestive heart failure
G5802	Decompensated cardiac failure
G5803	Compensated cardiac failure
G5804	Congestive heart failure due to valvular disease
G581.	Left ventricular failure
G5810	Acute left ventricular failure
G582.	Acute heart failure

For peer review only

G583.	Heart failure with normal ejection fraction
G584.	Right ventricular failure
G58z.	Heart failure NOS
**PAD codes**	**Description**
G73..	Other peripheral vascular disease
G734.	Peripheral arterial disease
G73y.	Other specified peripheral vascular disease
G73z.	Peripheral vascular disease NOS
G73z0	Intermittent claudication
G73zz	Peripheral vascular disease NOS
Gyu74	[X]Other specified peripheral vascular diseases
**Stroke codes**	**Description**
Fyu55	[X]Other transient cerebral ischaemic attacks and related syndromes
G61..	Intracerebral haemorrhage
G610.	Cortical haemorrhage
G611.	Internal capsule haemorrhage
G612.	Basal nucleus haemorrhage
G613.	Cerebellar haemorrhage
G614.	Pontine haemorrhage
G615.	Bulbar haemorrhage
G616.	External capsule haemorrhage
G618.	Intracerebral haemorrhage, multiple localized
G619.	Lobar cerebral haemorrhage
G61X.	Intracerebral haemorrhage in hemisphere, unspecified
G61X0	Left sided intracerebral haemorrhage, unspecified
G61X1	Right sided intracerebral haemorrhage, unspecified
G61z.	Intracerebral haemorrhage NOS
G63y0	Cerebral infarct due to thrombosis of precerebral arteries
G63y1	Cerebral infarction due to embolism of precerebral arteries
G64..	Cerebral arterial occlusion
G640.	Cerebral thrombosis
G6400	Cerebral infarction due to thrombosis of cerebral arteries
G641.	Cerebral embolism
G6410	Cerebral infarction due to embolism of cerebral arteries
G64z.	Cerebral infarction NOS
G64z0	Brainstem infarction
G64z1	Wallenberg syndrome
G64z2	Left sided cerebral infarction

For peer review only

G64z3	Right sided cerebral infarction
G64z4	Infarction of basal ganglia
G65..	Transient cerebral ischaemia
G650.	Basilar artery syndrome
G651.	Vertebral artery syndrome
G6510	Vertebro-basilar artery syndrome
G652.	Subclavian steal syndrome
G653.	Carotid artery syndrome hemispheric
G654.	Multiple and bilateral precerebral artery syndromes
G656.	Vertebrobasilar insufficiency
G657.	Carotid territory transient ischaemic attack
G65y.	Other transient cerebral ischaemia
G65z.	Transient cerebral ischaemia NOS
G65z0	Impending cerebral ischaemia
G65z1	Intermittent cerebral ischaemia
G65zz	Transient cerebral ischaemia NOS
G66..	Stroke and cerebrovascular accident unspecified
G660.	Middle cerebral artery syndrome
G661.	Anterior cerebral artery syndrome
G662.	Posterior cerebral artery syndrome
G663.	Brain stem stroke syndrome
G664.	Cerebellar stroke syndrome
G665.	Pure motor lacunar syndrome
G666.	Pure sensory lacunar syndrome
G667.	Left sided CVA
G668.	Right sided CVA
G6760	Cerebral infarction due to cerebral venous thrombosis, nonpyogenic
G6W..	Cerebral infarction due to unspecified occlusion or stenosis of precerebral arteries
G6X..	Cerebral infarction due to unspecified occlusion or stenosis of cerebral arteries
Gyu62	[X]Other intracerebral haemorrhage
Gyu63	[X]Cerebral infarction due to unspecified occlusion or stenosis of cerebral arteries
Gyu64	[X]Other cerebral infarction
Gyu65	[X]Occlusion and stenosis of other precerebral arteries
Gyu66	[X]Occlusion and stenosis of other cerebral arteries
Gyu6F	[X]Intracerebral haemorrhage in hemisphere, unspecified
Gyu6G	[X]Cerebral infarction due to unspecified occlusion or stenosis of precerebral arteries
ZV12D	[V]Personal history of transient ischaemic attack

For peer review only

Supplementary Table 2 - ICD-10 chapters used to identify admissions for cardiovascular disease

ICD-10 chapter	Description
I20	Angina pectoris
I21	Acute myocardial infarction
I22	Subsequent myocardial infarction
I23	Certain current complications following acute myocardial infarction
I24	Other acute ischaemic heart diseases
I25	Chronic ischaemic heart disease
I26	Pulmonary embolism
I27	Other pulmonary heart diseases
I28	Other diseases of pulmonary vessels
I30	Acute pericarditis
I31	Other diseases of pericardium
I32	Pericarditis in diseases classified elsewhere
I33	Acute and subacute endocarditis
I34	Nonrheumatic mitral valve disorders
I35	Nonrheumatic aortic valve disorders
I36	Nonrheumatic tricuspid valve disorders
I37	Pulmonary valve disorders
I38	Endocarditis, valve unspecified
I39	Endocarditis and heart valve disorders in diseases classified elsewhere
I40	Acute myocarditis
I41	Myocarditis in diseases classified elsewhere
I42	Cardiomyopathy
I43	Cardiomyopathy in diseases classified elsewhere
I44	Atrioventricular and left bundle-branch block
I45	Other conduction disorders
I46	Cardiac arrest
I47	Paroxysmal tachycardia
I48	Atrial fibrillation and flutter
I49	Other cardiac arrhythmias
I50	Heart failure
I51	Complications and ill-defined descriptions of heart disease
I52	Other heart disorders in diseases classified elsewhere
I60	Subarachnoid haemorrhage
I61	Intracerebral haemorrhage
I62	Other nontraumatic intracranial haemorrhage
I63	Cerebral infarction
I64	Stroke, not specified as haemorrhage or infarction

For peer review only

I65	Occlusion and stenosis of precerebral arteries, not resulting in cerebral infarction
I66	Occlusion and stenosis of cerebral arteries, not resulting in cerebral infarction
I67	Other cerebrovascular diseases
I68	Cerebrovascular disorders in diseases classified elsewhere
I69	Sequelae of cerebrovascular disease

Supplementary Table 3 - Variables included in the CPRD analysis

Variable	Categories
Age	20-39, 40-59, 60-79, ≥80
Sex	Male, Female
Index of Multiple Deprivation	Quintiles
Coronary heart disease	Yes (at anytime in medical history), No
Heart failure	Yes (at anytime in medical history), No
Peripheral arterial disease	(at anytime in medical history)
Stroke	Stroke (at anytime in medical history), Transient Ischaemic Attack (at anytime in medical history but no stroke), No

Supplementary Table 4 - Univariable changes over time in CPRD (by mid-year)

	2001	2005	2010	2015
**Number of patients**	1,122,722	1,840,798	2,207,027	1,340,441
**Age (%)**				
20-39	31.6	31.2	31.9	31.5
40-59	41.1	38.9	36.8	36.8
60-79	23.8	24.2	24.5	24.8
≥80	3.4	5.7	6.7	6.9
**Female (%)**	50.9	50.6	50.6	50.8
**Index of Multiple Deprivation (mean)**	2.72	2.74	2.75	2.75
**Coronary heart disease (%)**	4.5	4.9	4.5	4.0
**Heart failure (%)**	0.7	1.0	1.0	1.0
**Peripheral arterial disease (%)**	0.7	1.0	1.0	0.8

For peer review only

Stroke (%)				
Transient ischaemic attack	0.7	0.9	1.0	0.9
Stroke	0.8	1.2	1.4	1.4

Note: Data on Index of Multiple Deprivation was missing for <0.1% of patients. These patients were excluded from the analysis.

Supplementary Table 5 - Variables included in the HES analysis

Variable	Categories
Age	40-44, 45-49, 50-54, 55-59, 60-64, 65-69, 70-74, ≥75
Sex	Male, Female
Index of Multiple Deprivation	Quintiles
Cardiovascular code	3-character ICD-10 code ranging from I20 to I69

Note: admissions with more than one included cardiovascular code were counted multiple times

Supplementary Table 6 - Univariable changes over time in HES (by mid-year)

	2001	2005	2010	2015
**Number of admissions**	38861	38855	39318	42756
**Age (%)**				
40-44	3.06	3.76	3.31	2.85
45-49	4.79	5.46	5.66	5.26
50-54	8.74	7.75	8.09	8.47
55-59	11.29	11.66	10.53	11.01
60-64	13.93	13.73	15.33	13.02
65-69	16.76	16.66	16.07	18.19
70-74	18.85	18.70	18.71	18.23
≥75	22.58	22.25	22.27	22.92
**Female (%)**	36.21	35.95	35.66	35.26
**Index of Multiple Deprivation (mean)**	1.74	1.79	1.77	1.76
**Cardiovascular code (top 5 %)**				
I25 Chronic CHD	23.4	26.52	27.39	22.94

For peer review only

I20 Angina pectoris	19.8	15.74	11.97	7.75
I48 Atrial fibrillation	11.29	12.72	15.56	16.85
I21 Acute myocardial infarction	10.53	10.21	6.85	11.40
I50 Heart failure	8.33	6.81	5.98	7.39

Note: There was no missing data

For peer review only

Supplementary Figure 1 - Number of primary care practice included in the analysis by month

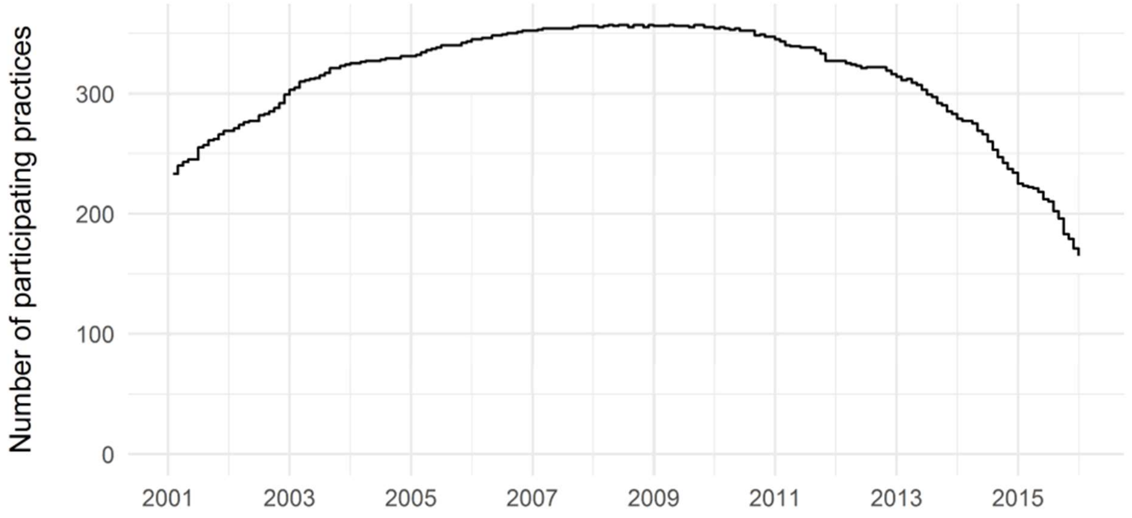

Supplementary Figure 2 - Information-Geometric Temporal (IGT) plot of demography (including age) and cardiovascular disease prevalence in CPRD between 2001 and 2015. The observed vertical columns are due to the way CPRD records age (birth year only), so changes in age can only happen in July.

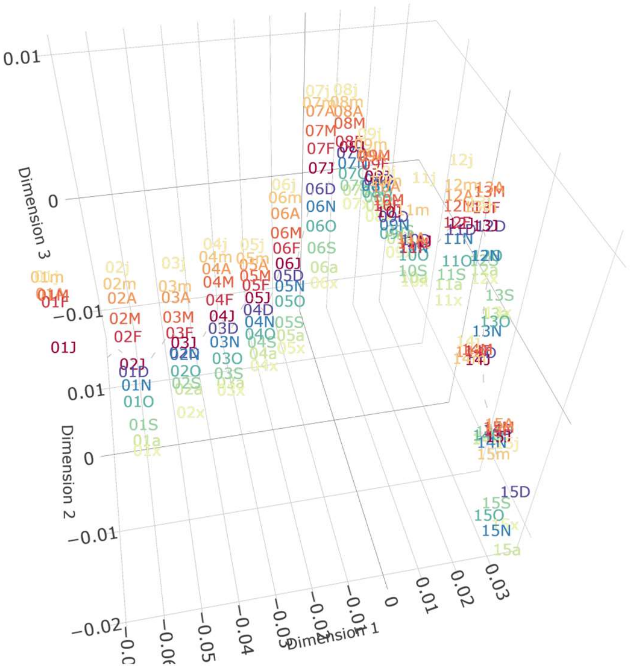

Supplementary Figure 3 - Information-Geometric Temporal (IGT) subplots of Figure 2
(a) Between 2001-2008 there was a gradual increase in disease prevalence, with two indentations corresponding to the years 2003 and 2005. (b) In 2008, the general trend reverses and prevalences decrease again, shown by a change in the direction of the graph. (c) The magnitude of variability increases after 2011, predominantly owing to changes in the socio-economic status due to a reduction in the number of practices contributing to the dataset.

(a)

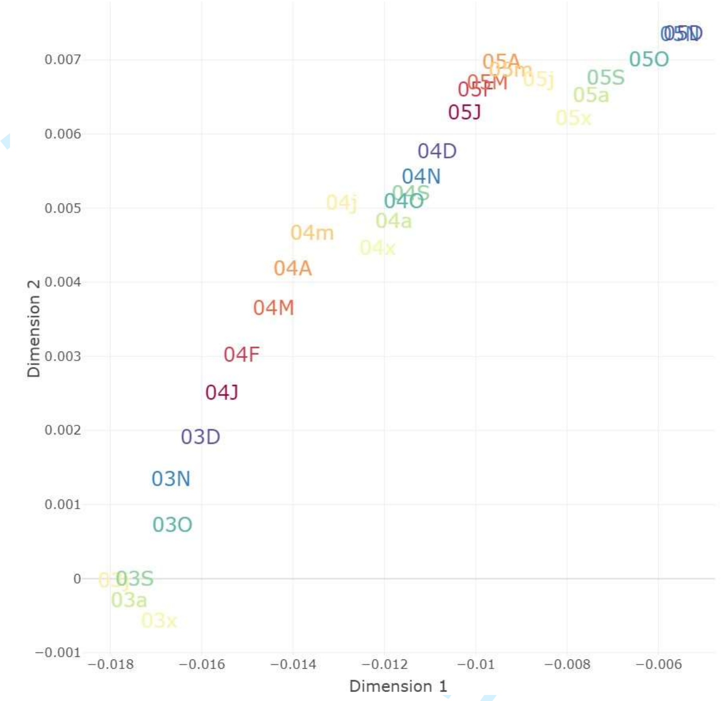

w only

(b)

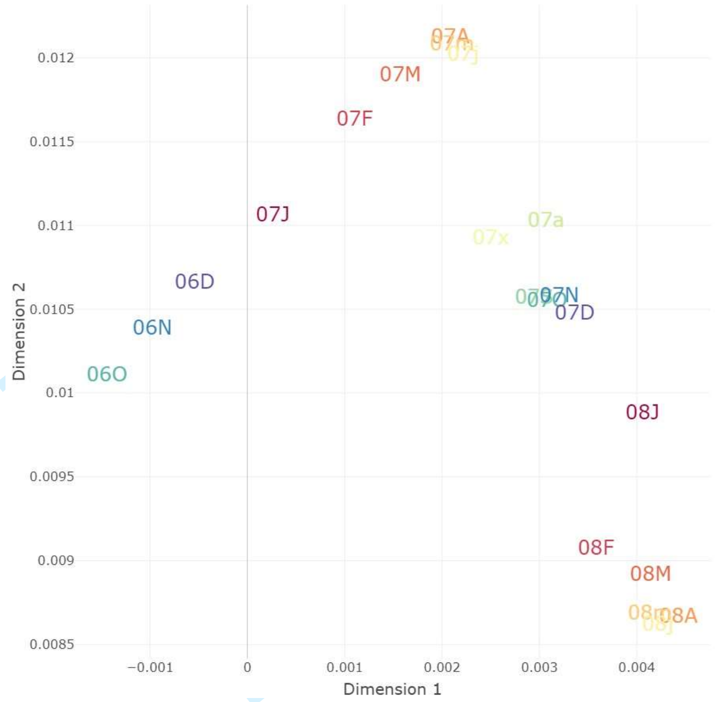
07A
07M
07F
07J
07a
07x
06D
07N
07D
06N
06O
08J
08F
08M
08A
0.012
0.0115
0.011
0.0105
0.01
0.0095
0.009
0.0085
−0.001
0
0.001
0.002
0.003
0.004
Dimension 1
Dimension 2

(c)

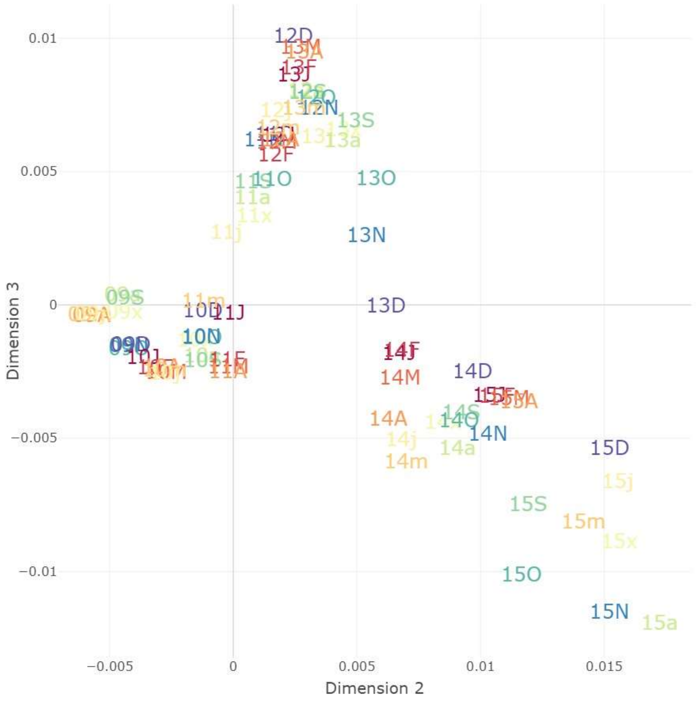
12D
13S
13a
13O
13N
13D
09S
10D
11J
10N
11F
14F
14J
14M
14D
14A
14O
14N
14a
14m
14j
15D
15j
15S
15m
15x
15O
15N
15a
11O
11a
11x
11j
12F
0.01
0.005
0
−0.005
−0.01
0.015
Dimension 2
Dimension 3

Supplementary Figure 4 - Information-Geometric Temporal (IGT) subplots of Figure 3
(a) From 2001-2009 there was a gradual change in which cardiovascular codes were associated with hospital admission. The data distributions starts to diverge from the previous trend in March 2009. (b) In March 2010, the distribution of cardiovascular codes abruptly changes. (c and d) Similar and even stronger changes in cardiovascular disease coding occurred again in April 2012 and April 2014.

(a)

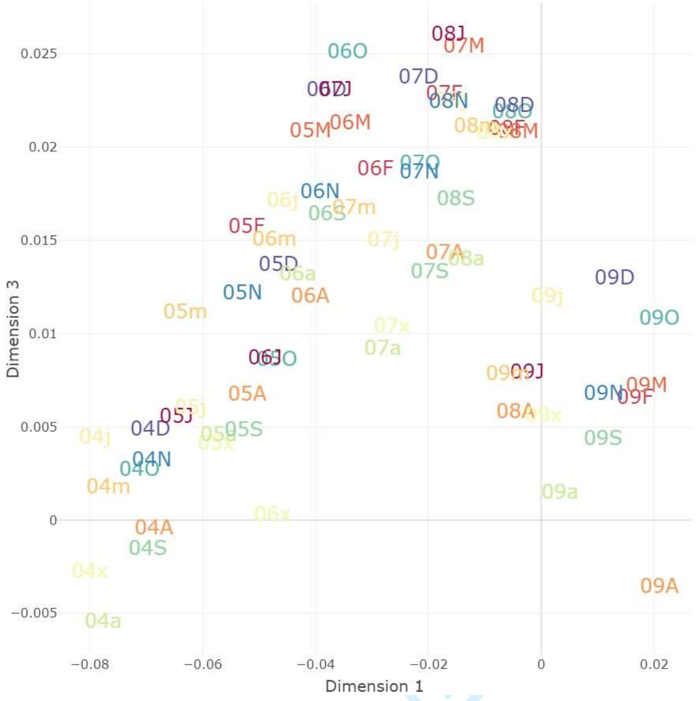

(b)

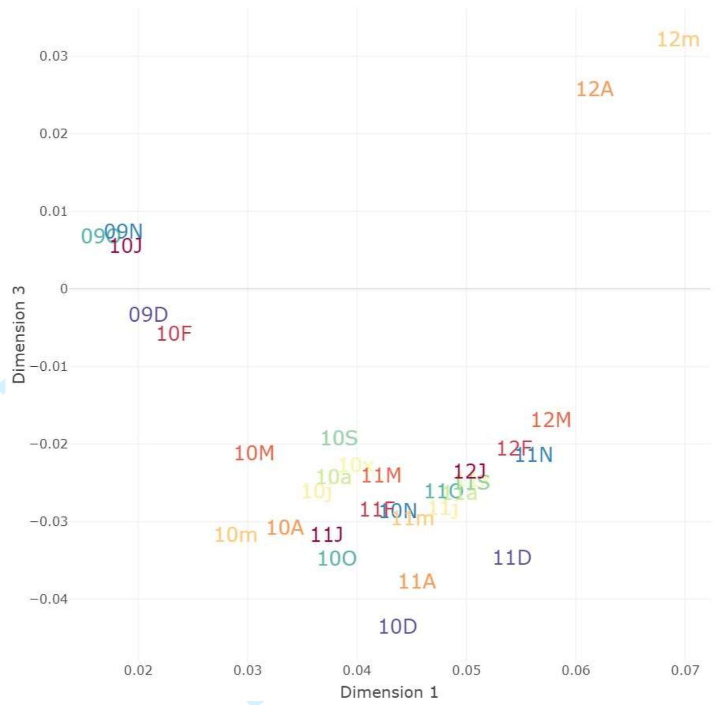
12m
12A
09N
10J
09D
10F
12M
10S
12F
11N
10M
11M
12J
10a
10j
11S
11F
10N
10m
10A
11J
10O
11D
11A
10D
Dimension 3
Dimension 1

(c)

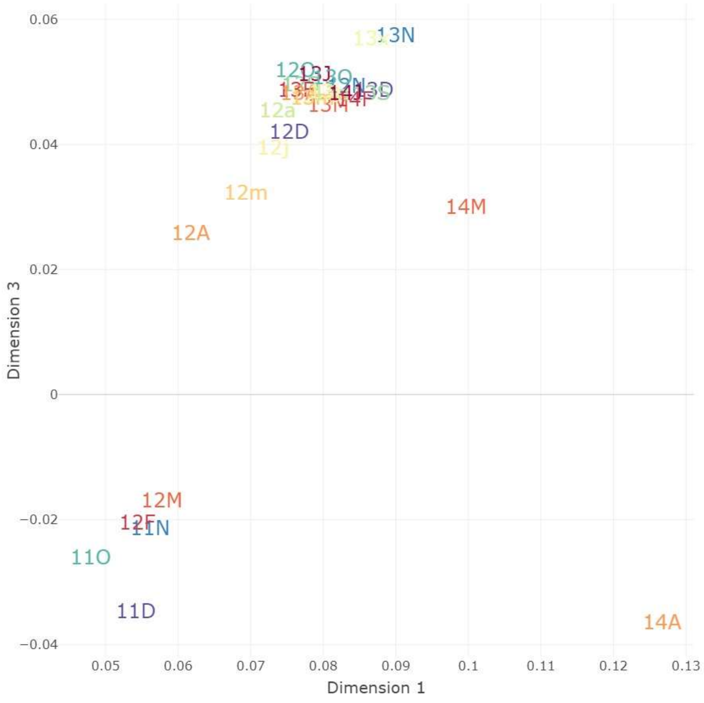
13N
12O
13J
13F
12a
13M
12D
12j
12m
14M
12A
12M
12F
11N
11O
11D
14A
Dimension 3
Dimension 1

(d)

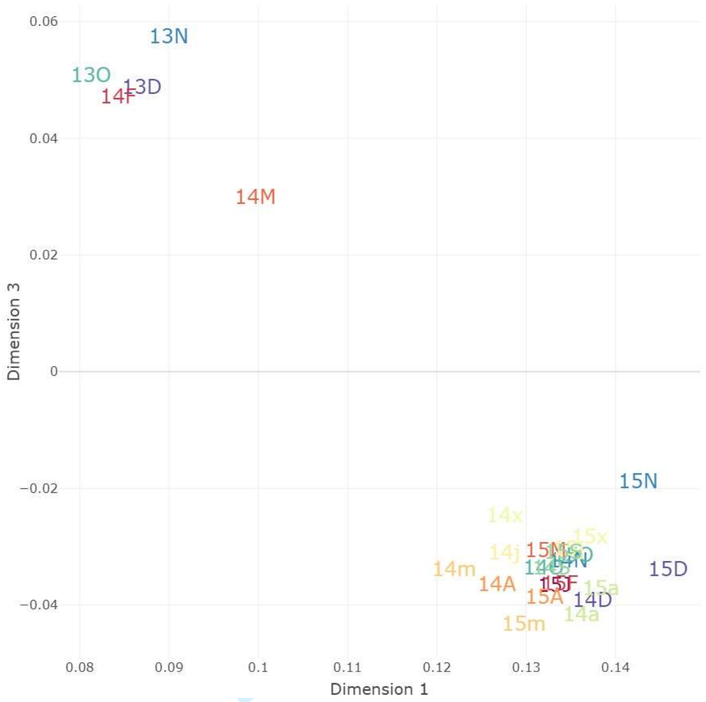

For peer review only

The RECORD statement – checklist of items, extended from the STROBE statement, that should be reported in observational studies using routinely collected health data.

	Item No.	STROBE items	Location in manuscript where items are reported	RECORD items	Location in manuscript where items are reported
**Title and abstract**					
	1	(a) Indicate the study's design with a commonly used term in the title or the abstract (b) Provide in the abstract an informative and balanced summary of what was done and what was found	(a) This study contains a non-standard study design. It is therefore titled as a case study on EHR.<br><br>(b) Abstract created in the format required by BMJ Open.	RECORD 1.1: The type of data used should be specified in the title or abstract. When possible, the name of the databases used should be included.<br><br>RECORD 1.2: If applicable, the geographic region and timeframe within which the study took place should be reported in the title or abstract.<br><br>RECORD 1.3: If linkage between databases was conducted for the study, this should be clearly stated in the title or abstract.	1.1. Electronic health records databases mentioned in the title<br><br>1.2. Geography and time included<br><br>1.3. No linkage included.
**Introduction**					
Background rationale	2	Explain the scientific background and rationale for the investigation being reported	Introduction		
Objectives	3	State specific objectives, including any prespecified hypotheses	Introduction – paragraph		
**Methods**					
Study Design	4	Present key elements of study design early in the paper	The methods section first introduces the database, then defines the inclusion criteria, then explains the methods		

For peer review only

			used and finally discusses the specific statistical analysis.		
Setting	5	Describe the setting, locations, and relevant dates, including periods of recruitment, exposure, follow-up, and data collection	Methods – Data Sources<br>Methods – Study design and population		
Participants	6	*(a) Cohort study* - Give the eligibility criteria, and the sources and methods of selection of participants. Describe methods of follow-up<br>*Case-control study* - Give the eligibility criteria, and the sources and methods of case ascertainment and control selection. Give the rationale for the choice of cases and controls<br>*Cross-sectional study* - Give the eligibility criteria, and the sources and methods of selection of participants<br><br>*(b) Cohort study* - For matched studies, give matching criteria and number of exposed and unexposed<br>*Case-control study* - For matched studies, give matching criteria and the number of controls per case	(a)<br>Methods – Study design and population<br><br>(b)<br>No matching involved	RECORD 6.1: The methods of study population selection (such as codes or algorithms used to identify subjects) should be listed in detail. If this is not possible, an explanation should be provided.<br><br>RECORD 6.2: Any validation studies of the codes or algorithms used to select the population should be referenced. If validation was conducted for this study and not published elsewhere, detailed methods and results should be provided.<br><br>RECORD 6.3: If the study involved linkage of databases, consider use of a flow diagram or other graphical display to demonstrate the data linkage process, including the number of individuals with linked data at each stage.	6.1<br>Methods – Study design and population<br><br>Code lists separately included in the supplement<br><br>6.2<br>This is a validation study.<br><br>6.3<br>No data linkage involved.
Variables	7	Clearly define all outcomes, exposures, predictors, potential confounders, and effect modifiers. Give diagnostic criteria, if applicable.	Methods – temporal variability metrics	RECORD 7.1: A complete list of codes and algorithms used to classify exposures, outcomes, confounders, and effect modifiers should be provided. If	7.1.<br>Code lists separately included in the supplement

For peer review only

				these cannot be reported, an explanation should be provided.	
Data sources/ measurement	8	For each variable of interest, give sources of data and details of methods of assessment (measurement).<br>Describe comparability of assessment methods if there is more than one group	Methods – data sources		
Bias	9	Describe any efforts to address potential sources of bias	This is a validation study intended to identify bias.		
Study size	10	Explain how the study size was arrived at	Study size is secondary for this analysis. Numbers and descriptive statistics are provided in the supplement.		
Quantitative variables	11	Explain how quantitative variables were handled in the analyses. If applicable, describe which groupings were chosen, and why	Methods – Study design and population		
Statistical methods	12	(a) Describe all statistical methods, including those used to control for confounding<br>(b) Describe any methods used to examine subgroups and interactions<br>(c) Explain how missing data were addressed<br>(d) *Cohort study* - If applicable, explain how loss to follow-up was addressed<br>*Case-control study* - If applicable, explain how	(a)<br>Methods – temporal variability metrics<br>Methods – statistical analysis<br><br>(b)<br>Methods – statistical analysis<br><br>(c)<br>No missing data was present		

For peer review only - http://bmjopen.bmj.com/site/about/guidelines.xhtml

		matching of cases and controls was addressed<br>*Cross-sectional study* - If applicable, describe analytical methods taking account of sampling strategy<br>(e) Describe any sensitivity analyses	(d)<br>Not applicable<br><br>(e)<br>No sensitivity analysis was performed.		
Data access and cleaning methods		..		RECORD 12.1: Authors should describe the extent to which the investigators had access to the database population used to create the study population.<br><br>RECORD 12.2: Authors should provide information on the data cleaning methods used in the study.	12.1<br>Data sharing statement<br><br>12.2<br>Code provided on Github
Linkage		..		RECORD 12.3: State whether the study included person-level, institutional-level, or other data linkage across two or more databases. The methods of linkage and methods of linkage quality evaluation should be provided.	12.3<br>No linkage involved.
**Results**					
Participants	13	(a) Report the numbers of individuals at each stage of the study (*e.g.*, numbers potentially eligible, examined for eligibility, confirmed eligible, included in the study, completing follow-up, and analysed)<br>(b) Give reasons for non-participation at each stage.<br>(c) Consider use of a flow diagram	(a)<br>Numbers provided in the supplement. Not relevant to the main argument or interpretation of the study results.<br><br>(b)<br>Inclusion was solely based on registration with the GP, as described in	RECORD 13.1: Describe in detail the selection of the persons included in the study (*i.e.,* study population selection) including filtering based on data quality, data availability and linkage. The selection of included persons can be described in the text and/or by means of the study flow diagram.	13.1<br>Methods – Study design and population<br><br>Code provided on Github

For peer review only

			Methods – Study design and population<br><br>(c)<br>Study population changes throughout the study, flow diagram not appropriate.		
Descriptive data	14	(a) Give characteristics of study participants (*e.g.*, demographic, clinical, social) and information on exposures and potential confounders<br>(b) Indicate the number of participants with missing data for each variable of interest<br>(c) *Cohort study* - summarise follow-up time (*e.g.*, average and total amount)	(a)<br>Supplementary material<br><br>(b)<br>Only socio-economic status could be missing by definition.<br>Methods – Study design and population<br><br>(c)<br>Not applicable		
Outcome data	15	*Cohort study* - Report numbers of outcome events or summary measures over time<br>*Case-control study* - Report numbers in each exposure category, or summary measures of exposure<br>*Cross-sectional study* - Report numbers of outcome events or summary measures	Not applicable – there is no outcome event		
Main results	16	(a) Give unadjusted estimates and, if applicable, confounder-	(a)<br>Not applicable		

For peer review only

		adjusted estimates and their precision (e.g., 95% confidence interval). Make clear which confounders were adjusted for and why they were included<br>(b) Report category boundaries when continuous variables were categorized<br>(c) If relevant, consider translating estimates of relative risk into absolute risk for a meaningful time period	(b)<br>Methods – Study design and population<br><br>(c)<br>Not applicable		
Other analyses	17	Report other analyses done—e.g., analyses of subgroups and interactions, and sensitivity analyses	No additional sensitivity analysis was performed		
**Discussion**					
Key results	18	Summarise key results with reference to study objectives	Discussion – paragraph 1		
Limitations	19	Discuss limitations of the study, taking into account sources of potential bias or imprecision. Discuss both direction and magnitude of any potential bias	Discussion - Limitations	RECORD 19.1: Discuss the implications of using data that were not created or collected to answer the specific research question(s). Include discussion of misclassification bias, unmeasured confounding, missing data, and changing eligibility over time, as they pertain to the study being reported.	19.1<br>This is the main topic of this analysis
Interpretation	20	Give a cautious overall interpretation of results considering objectives, limitations, multiplicity of analyses, results from similar studies, and other relevant evidence	Discussion – Limitations<br>Discussion – paragraphs 2-4		
Generalisability	21	Discuss the generalisability (external validity) of the study results	Discussion - Limitations		

Other Information					
Funding	22	Give the source of funding and the role of the funders for the present study and, if applicable, for the original study on which the present article is based	Funding statement		
Accessibility of protocol, raw data, and programming code		..		RECORD 22.1: Authors should provide information on how to access any supplemental information such as the study protocol, raw data, or programming code.	Data sharing statement<br><br>Code provided on Github

*Reference: Benchimol EI, Smeeth L, Guttmann A, Harron K, Moher D, Petersen I, Sørensen HT, von Elm E, Langan SM, the RECORD Working Committee. The REporting of studies Conducted using Observational Routinely-collected health Data (RECORD) Statement. *PLoS Medicine* 2015; in press.

*Checklist is protected under Creative Commons Attribution (CC BY) license.

For peer review only
